# A strategy to re-sensitise drug-resistant Gram-positive bacteria to oxazolidinone-class antibiotics

**DOI:** 10.1016/j.ebiom.2025.105914

**Published:** 2025-09-04

**Authors:** Qi Zhang, Yang Yang, Ying Yang, Jin Shang, Shan Su, Peng Gao, Xiao-Xiao Li, Zhao Liu, Richard Yi-Tsun Kao, Ben Chi-Bun Ko, Benjamin Thompson, Qian Zhao

**Affiliations:** a State Key Laboratory of Chemical Biology and Drug Discovery, Department of Applied Biology and Chemical Technology, The Hong Kong Polytechnic University, Hong Kong; b Centre for Eye and Vision Research, Hong Kong; c State Key Laboratory of Chinese Medicine and Molecular Pharmacology (Incubation), Shenzhen Research Institute, The Hong Kong Polytechnic University, Shenzhen, China; d Applied Oral Sciences and Community Dental Care, Faculty of Dentistry, The University of Hong Kong, Sassoon Road, Hong Kong; e Department of Microbiology, The University of Hong Kong, Sassoon Road, Hong Kong; f School of Optometry and Vision Science, University of Waterloo, Waterloo, ON, Canada

**Keywords:** Antimicrobial resistance, Phosphorylated prodrugs, Oxazolidinone-class antibiotics, Tedizolid phosphate, Lysozyme, Ocular microbiome

## Abstract

**Background:**

Multidrug-resistant bacterial infections have high mortality rates and few treatment options. Synergistic combinations may improve clinical outcome but traditional strategies often damage healthy microbiome. Oxazolidinone-class antibiotics are typical last-resort drugs for treating drug-resistant bacterial infections but are becoming less effective due to resistance development.

**Methods:**

After high-throughput screening, synergy was further assessed by *in vitro* indices (like fractional inhibitory concentration index, biofilm formation and resistance development) and *in vivo* symptoms in animals with skin and ocular bacterial infections (and ocular microbiome extraction analysis). Proteomics, chemical synthesis, multi-microscopy techniques and antibiotic real-time/kinetic accumulation were employed to explore mechanisms and expand translational applications.

**Findings:**

Combining phosphorylated oxazolidinone-class antibiotics with positively charged compounds (lysozyme as native representative) resulted in broad-spectrum drug re-sensitisation. In representative combination, urea cycle was disrupted to alkalinise cytoplasm, which subsequently activated alkaline phosphatase to promote conversion of phosphorylated prodrug to active form. By introducing concept of restored healthy microbiome as the evaluated index in antibiotic therapy, we confirmed excellent translational and microbiome-friendly potential of this strategy in clinical settings because it not only inhibited biofilm formation and development of drug-resistant mutations *in vitro*, but also alleviated symptoms in infected animals including the restoration of healthy microbiome.

**Interpretation:**

As both agents have excellent safety profiles, such clinical investigation may immediately be contemplated in humans. Translationally, scientists benefit from strategy by simultaneously achieving greater efficacy (>500-fold re-sensitisation) and higher safety (prodrug-based and microbiome-friendly strategy especially when active form may be toxic).

**Funding:**

Collaborative Research Funds from 10.13039/501100002920Research Grants Council (C5033-19E).

**Research in context**

**Evidence before this study**

Antimicrobial resistance (AMR) and AMR-associated superbugs represent a global health crisis. Among these lethal “*ESKAPE*” pathogens prioritised by the World Health Organization (WHO), drug-resistant Gram-positive bacteria, *i.e., Staphylococcus aureus* (and *Enterococcus faecium*), are associated with the high incidence of both community-acquired and hospital-acquired infections, which carry significant mortality rates. The oxazolidinone-class antibiotics like linezolid exhibit broad-spectrum efficacy through versatile administration, making them a last-resort drug for treating drug-resistant Gram-positive bacterial infections. However, their effectiveness is diminishing due to AMR and few solutions. To preserve effectiveness of certain antibiotic, combination therapy of this antibiotic and its adjuvant might be deserved to receive attention. Ideally, this approach can kill drug-resistant pathogen with lower doses of antibiotics, thereby slowing down the emergence of AMR-associated superbugs. However, traditional antimicrobial (combination) therapy often significantly disrupted the healthy microbiome (an emerging concern in the antibiotic treatment), leading to various side-effects.

**Added value of this study**

After diverse evaluation of indices both *in vitro* and *in vivo*, three promising combination therapies (>500-fold re-sensitisation) were reported to combat broad-spectrum drug-resistant Gram-positive bacteria. On this basis, a translatable and microbiome-friendly strategy was further developed to restore the activity of many oxazolidinone-class antibiotics (as last-resort drugs) by combining phosphorylated prodrugs with lysozyme (as a representative adjuvant), in which a concept of restored healthy microbiome was firstly introduced as evaluated index, accompanied by comprehensive ocular microbiome reports of healthy (and infected) C57BL/6 mice under antibiotic therapy. Meanwhile, there were few reports on such a synergistic mechanism, in which cost-effective lysozyme works as adjuvant (instead of its well-known role as antimicrobial agent) enhanced the bactericidal efficacy of tedizolid phosphate by disrupting the urea cycle, which synergistically destroys pH homoeostasis with the help of the fragile homoeostasis caused by lysozyme with a high positive charge. This disruption subsequently activates the alkaline phosphatase and accelerates the conversion of the phosphorylated prodrug to its active form, thereby boosting its effective concentration in bacteria. Multi-omics approaches have further facilitated interdisciplinary findings, including the identification of the roles of tedizolid phosphate in energy metabolism and uneven cellular division through proteomics and multi-microscopy techniques, as well as the profiling of two newly synthesised phosphorylated oxazolidinone-class antibiotics.

**Implications of all the available evidence**

Beside lysozyme with excellent safety profile, tedizolid phosphate is also widely used to treat multidrug-resistant Gram-positive bacterial infections, particularly in acute bacterial skin and skin structure infections on tissues containing diverse microbiome. Thus, clinical studies could immediately be contemplated for patients suffering these infections on tissues including skin and eye, with a potential to reduce unacceptably high morbidity, blinding and even mortality rates. However, such clinical evaluation in humans is limited by the narrowness of the available testing paradigms due to the pending approval status of other oxazolidinone-class antibiotics including delpazolid and eperezolid. Notably, regarding drug with potential toxicity, such as tedizolid causing potential peripheral neuropathy, its safety is often enhanced by commercialising its phosphorylated prodrug (tedizolid phosphate) in the pharmaceutical industry at the expense of its efficacy. Our strategy can simultaneously achieve greater efficacy while improving safety. Given the conservation and cost-effectiveness of lysozyme, this strategy should be vigorously promoted to the pharmaceutical industry in the future.

## Introduction

In the post-antibiotic era, antibiotics, albeit as the primary treatment for bacterial infections, are facing challenges such as drug-resistance and disruption of healthy microbiome.^1^ This AMR and microbiome imbalance significantly reduced their clinical efficacies and raised the risk of recurrent infections, posing a serious threat to global health.^2^

The oxazolidinone-class antibiotics are one of the newest classes of antibacterial drugs, which have clear pharmacokinetic and pharmacodynamics characteristics, and inhibit protein synthesis by targeting bacterial 23S rRNA.^3^ Compared with other antibiotics, oxazolidinone-class antibiotics, like linezolid, can exhibit broad-spectrum antibacterial efficacies by versatile administration,^3^ making them a last-resort drug for treating drug-resistant Gram-positive bacterial infections. Among these oxazolidinone-class antibiotics, tedizolid phosphate (TP, Sivextro®) is FDA-approved at 2014 and widely used to treat multidrug-resistant Gram-positive bacterial infections, particularly in acute bacterial infections affecting tissues such as the skin, which contains a diverse microbiome.^4^, ^5^, ^6^ TP as prodrug is converted into its active form, tedizolid (T), by alkaline phosphatase (ALP) *in vivo*.^4^^,^^5^ This conversion facilitates targeted therapy effects on sensitive and insensitive tissues, as the efficiency of ALP to remove the phosphate group in different tissues influences the intracellular concentrations of T. Notably, such prodrug with phosphorylation modification is widely applied in the pharmaceutical industry due to its advantages such as reduced toxicity, enhanced bio-stability and improved lipid-solubility for efficient cell membrane penetration.^7^^,^^8^ However, due to overuse or misuse, oxazolidinone-class antibiotics including TP and linezolid are becoming ineffective in clinical settings.

To preserve the effectiveness of certain antibiotics, combination therapy of this antibiotic and its adjuvant (as a resistance breaker) has been recognised as a safer, more economical and effective alternative.^2^^,^^9^ Ideally, this approach can kill pathogens with lower doses of antibiotics, thereby slowing down the emergence of AMR-associated superbugs.^2^ However, traditional antimicrobial (combination) therapy often significantly damaged the healthy microbiome, leading to various side-effects. Due to microbiome-friendly properties^10^ and safety profiles,^11^ natural bio-molecules such as cellular metabolites should be favourable adjuvants to antibiotics, which can significantly reduce required dosages of antibiotics through synergistic effects, minimising the damage to the healthy microbiome as much as possible.^2^^,^^9^^,^^12^ However, the concept is still stalled, which may be attributed to its interdisciplinary nature as it relates to the latest research frontiers.

Both the skin and ocular surface typically sustain distinct microbiome composed of various microorganisms.^13^^,^^14^ However, due to exposure to environmental contaminants, compared with others,^14^ these regions are more susceptible to infections throughout our lives, sometimes leading to recurrent infections.^15^^,^^16^ Interestingly, both skin and eye are protected by various physiological mechanisms, with lysozyme secretion being the most significant. As a macro-molecular cellular metabolite, lysozyme is synthesised by epithelial cells, glandular cells and white blood cells on a large scale, and widely secreted into skin and bio-fluids including blood, saliva, tears and breast milk to protect us from bacterial infection (>1 mg/ml in adults' tears on average).^17^ Compared to small cellular metabolites like glucose,^18^ lysozyme exhibits specific bacterial-targeting and broad applicability across different patients, leading to its great universality and bio-compatibility in different clinical practices.^17^ Importantly, this metabolite also evolves into a non-immunogenic enzyme in immune regulation for wound-healing.^19^ However, due to the mutations in genes such as *oatA*,^17^^,^^20^ lysozyme, albeit as the well-known antibacterial enzyme, doesn't combat the certain pathogenic infections, weakening its advantages in practical applications. Interestingly, due to its rich positive charges under physiological conditions,^17^^,^^21^ this safe and cost-effective macro-molecular metabolite may be also defined as a homoeostasis-breaker (adjuvant to antibiotic, instead of antimicrobial agent), which is neglected for a long period and fewer reports are available.

In this study, we firstly-evaluated the potential of native metabolite, especially lysozyme, as adjuvant to thousands of FDA-approved antibiotics and reported a practical and cost-effective strategy to restore activities of various oxazolidinone-class antibiotics by utilising their phosphorylated prodrugs. Mechanistic study on representative combination revealed that the intracellular levels of oxazolidinone-class antibiotic-derived compounds (phosphorylated prodrugs and their active forms) were enhanced when combined with positively charged compounds (lysozyme as a native representative adjuvant of TP, which was independent of its well-known role as antimicrobial agent). Importantly, by firstly introducing the conceptual index of whether the healthy microbiome was restored to evaluate the effectiveness of antibiotic treatments in infected tissues, we confirmed that a translatable and microbiome-friendly strategy had been developed to combat bacterial infections resistant to oxazolidinone-class antibiotics, with demonstrated efficacy in both skin and ocular infection models *in vivo*. Our study addresses the gap in understanding about how to reverse the resistance phenotypes to oxazolidinone-class antibiotics while maximising the protection of a healthy microbiome in applied regions.

## Methods

### High-throughput screening and checkerboard microdilution assays

A library of 1953 FDA-approved compounds (2.5 μM each, Table S1) was screened against methicillin-resistant *Staphylococcus aureus* USA300 (*MRSA*) or *Staphylococcus epidermidis* (*MRSE*) (1 × 10^6^ colony forming units (CFUs) per millilitre (CFU/ml)) in Luria–Bertani (LB) medium with or without lysozyme (1.0 mg/ml). Growth was monitored hourly for 24 h, with the absorbance at 600 nm (OD_600_) measured at endpoint. Inhibition ratios were calculated as: (OD_control_ − OD_sample_)/(OD_control_ − OD_background_) × 100%, where LB medium only and those included bacteria only served as background and control groups. Synergy was defined as a relative inhibition ratio of ≥90%.

According to the standard two-fold dilution method,^2^ TP (or other drugs) were serially diluted in fresh LB medium (or Trypticase Soy Broth medium supplemented with 5% defibrinated horse blood) containing various metabolites (NADH, pyruvate, etc.) in a 96-well plate. Each well was inoculated with 20.0 μl of 1.0 × 10^7^ CFU/ml logarithmic cultures of *MRSA* or *MRSE* and incubated overnight. Drug-free LB medium with or without bacteria served as the growth control and background control groups, respectively. The inhibition ratio (%) was calculated by measuring the OD_600_ of the bacterial culture using the formula: [1 − (OD_sample_ − OD_background_)/(OD_control_ − OD_background_)] × 100%. Bacterial viability was confirmed by serial dilution/plating. The minimum inhibitory concentration (MIC) was defined as the lowest concentration of the drug that inhibited 90% of microbial growth, as determined by both visual CFU counts and OD_600_ measurements (MIC_90_). The fractional inhibitory concentration (FIC) and the fractional inhibitory concentration index (FICI) were calculated as follows: FICI = (MIC_AB_/MIC_A_) + (MIC_BA_/MIC_B_) = FIC_A_ + FIC_B_,^22^ where MIC_A_ is the MIC of compound A alone, MIC_AB_ is the MIC of compound A in combination with compound B, MIC_B_ is the MIC of compound B alone, MIC_BA_ is the MIC of compound B in combination with compound A, FIC_A_ is the FIC of compound A in combination with compound B, and FIC_B_ is the FIC of compound B in combination with compound A. Synergistic, partially synergistic (additive), or non-synergistic effects are defined by FICI values of ≤0.5, 0.5 < FICI ≤ 1.0, or FICI > 1.0.

### Bactericidal effect testing

Overnight *MRSA* cultures were diluted 1:1000 and treated with TP (0.25 or 0.5 μg/ml), lysozyme (0.5 mg/ml), or their combination to construct time-killing curves at 37 °C. Bacterial loads were determined at time intervals of 0-, 1-, 3-, 6-, 9-, 12- and 22-h by serial dilution plating on LB agar (37 °C, 24 h). Biofilm formation was assessed using adherence assay on 96-well tissue culture plates. Briefly, *MRSA* (1.0 × 10^6^ CFU/ml) was treated with TP (0.5 μg/ml) in the presence or absence of lysozyme (0.5 mg/ml) for 24 h at 37 °C. After PBS washing, biofilms were methanol-fixed (95%, 60 °C, 15 min), crystal violet-stained (1%, 37 °C, 15 min), and solubilised with ethanol (95%) at 37 °C for 30 min. Biofilm strength was quantified at OD_590_ (strong: OD_sample_ ≥ 2OD_control_; weak: OD_control_ < OD_sample_ < 2OD_control_; none: OD_sample_ ≤ OD_control_), where LB medium served as the control group.

Log-phase *MRSA* was passaged daily (1:1000) in LB medium containing increasing concentrations of TP and/or lysozyme (37 °C, 24 h). MIC values were determined every 24-h via serial dilution for 12 days, with fold-change in MIC calculated relative to baseline (the initial MIC of TP or lysozyme).

*MRSA* culture in the log phase (1.0 × 10^10^ CFU/ml) was plated on gradient agar (TP: 0.5, 1.0, 2.0, and 4.0 μg/ml; lysozyme: 0.5, 1.0, 2.0, and 4.0 mg/ml). Mutation frequency was calculated as the ratio of colony counts of treated group to those in the initial group after 48 h incubation at 37 °C. The mutation prevention concentration (MPC) was defined as the lowest combination concentration preventing colony formation, with the mutation prevention index (MPI) calculated as MPC/MIC.

### Proteomic study

According to the previously described method,^23^ *MRSA* at exponential growth phase was exposed to lysozyme (0.5 mg/ml), TP (0.5 or 40 μg/ml), or their combination for 1 h at 37 °C. Proteins were extracted via freeze-thaw cycles and sonication in lysis buffer (25 mM HEPES-Na, 150 mM NaCl, 0.1% NP40, 4.0 M urea, and 1 × protease inhibitor), quantified by BCA assay (Thermo Fisher, China), and digested with trypsin (5:1 ratio). Peptides were TMT-16^plex^ labelled and analysed by LC-MS/MS (Orbitrap Exploris 480 with UltiMate 3000 UPLC, C^18^ column, Thermo Fisher, China) using a 120-min gradient (300 nl/min). Mobile phases A and B consisted of 0.1% formic acid (FA) in water and 0.1% FA in 100% acetonitrile (MeCN), respectively. Mobile phase B was increased to 6% at 12 min, 20% at 82 min, 30% at 92 min, and 90% at 100 min, held for an additional 5 min. MS data were acquired in data-dependent acquisition mode (MS1: 60,000 resolutions; MS2: 15,000 resolutions) and searched against the UniProt database for *S. aureus* (20,330 entries, accessed 09/2019, MaxQuant version 1.5.8.2) with carbamidomethylation (fixed) and methionine oxidation/N-terminal acetylation (variable). Trypsin was specified as an enzyme, allowing for two missed cleavages. The false discovery rate (FDR) for peptide spectral matches and protein identifications was set at 1%. The maximum number of modifications per peptide was limited to three. Differentially expressed proteins were defined as FDR-adjusted *p* ≤ 0.05 and fold change (FC) values ≥ 1.5 (log_2_ FC ≥ 0.58 or log_2_ FC ≤ −0.58; FDR: 5%).

### Chemical synthesis of delpazolid phosphate and eperezolid phosphate

Delpazolid phosphate was synthesised by reacting delpazolid with di-tert-butyl N,N-diethylphosphoramidite in a 1:1 M ratio in tetrazole (CH_2_N_4_) under conditions of hydrogen peroxide (H_2_O_2_) and dimethylacetamide (DMA), followed by hydrolysis using trifluoroacetic acid (TFA) and dichloromethane (DCM). Similarly, eperezolid phosphate was prepared by combining eperezolid with dibenzyl N,N-diisopropylphosphoramidite in a 1:1 M ratio in CH_2_N_4_ under conditions of MeCN and meta-chloroperoxybenzoic acid (mCPBA). After 18 h of reaction at room temperature, eperezolid phosphate was obtained by treating the mixture with palladium on carbon (Pd/C) in methanol (MeOH). Both compounds were purified by silica gel chromatography (ethyl acetate/n-hexane was 1:4), yielding delpazolid phosphate (white to off-white solid, 78% yield, >97% purity) and eperezolid phosphate (white to off-white solid, 44% yield, >97% purity), with purity confirmed by nuclear magnetic resonance (NMR) and liquid chromatography-mass spectrometry (LC-MS).

Delpazolid phosphate: ^1^H NMR (400 MHz, D_2_O) δ 8.14 (s, ^1^H), 7.57 (dd, J = 13.2, 2.4 Hz, ^1^H), 7.41 (t, J = 8.8 Hz, ^1^H), 7.34–7.28 (m, ^1^H), 4.95 (s, ^1^H), 4.20 (t, J = 9.2 Hz, ^1^H), 4.11 (ddd, J = 11.8, 5.6, 2.8 Hz, ^1^H), 4.03–3.96 (m, ^2^H), 3.94–3.90 (m, ^2^H), 3.82 (q, J = 6.4 Hz, ^1^H), 3.59 (t, J = 6.8 Hz, ^1^H), 3.44–3.39 (m, ^2^H), 2.85 (s, ^3^H); Eperezolid phosphate: ^1^H NMR (400 MHz, D_2_O) δ 7.36 (d, J = 12.8 Hz, ^1^H), 7.14 (d, J = 5.2, ^2^H), 4.32 (s, ^2^H), 4.16 (t, J = 9.2 Hz, ^1^H), 3.77 (dd, J = 9.2, 5.6, ^1^H), 3.71–3.69 (m, ^2^H), 3.57 (t, J = 7.2 Hz, ^1^H), 3.53–3.50 (m, ^4^H), 3.05 (t, J = 5.2 Hz, ^4^H), 2.63–2.59 (m, ^3^H), 1.93 (s, ^3^H).

### Nascent protein profiling

Click-iT™ plus OPP Alexa Fluor™ 647 protein synthesis kit containing OPP (O-propargyl-puromycin) probe was used to evaluate the inhibition of nascent protein synthesis. Specifically, mid-log-phase *MRSA* cultures with OD_600_ of 0.4–0.6 were treated with lysozyme, TP or their combination at different concentrations for 1 h at 37 °C. These bacterial cultures served as related negative control group. The positive control group followed a similar protocol but included the addition of 20.0 μM OPP. Next, all cells were washed, fixed (3.7% formaldehyde), and permeabilized (0.5% Triton X-100) before Click-iT® OPP reaction. After 30 min of incubation in the absence of light at room temperature, the reaction cocktail was removed. Next, the fixed cells in a 96-well plate were washed with 200 μl per well of Click-iT® reaction rinse buffer, followed by further incubation with the 1 × HCS nuclear Mask™ blue stain working solution. After another 30 min of incubation in the absence of light at room temperature, the nuclear Mask™ blue stain working solution was removed. Finally, the cells were washed twice with cold PBS before fluorescent analysis, according to the individual excitation and emission maxima (Alexa Fluor® 647 picolyl azide (λ_ex_ = 650 nm, λ_em_ = 670 nm); nuclear Mask™ blue stain: (λ_ex_ = 350 nm, λ_em_ = 451 nm)). The degree of inhibition of nascent protein synthesis was expressed as the fluorescence ratio (Alexa Fluor®/nuclearMask™), presented as the average ± SD.

### Probe-based cytoplasmic pH evaluation

Mid-log-phase *MRSA* or *MRSE* was treated with lysozyme, TP, or their combination at varying concentrations alongside 10.0 μM BCECF-AM pH-sensitive probe (Beyotime Biotechnology, China), while untreated cells and LB medium with probe served as the control and background groups, respectively. After 1 h incubation at 37 °C, 200 μl of each sample was transferred to a white 96-well plate. Fluorescence was measured (λ_ex_: 488 nm; λ_em_: 550–600 nm) to assess cytoplasmic pH, as this probe did not emit a fluorescence signal unless BCECF-AM was absorbed and converted into BCECF within cytoplasm.

### Antimicrobial levels

The intracellular levels of antimicrobials were determined by LC-MS/MS analysis according to previous report.^24^, ^25^, ^26^ Briefly, log-phase *MRSA* were treated with TP (0.5 μg/ml) and varying lysozyme concentrations (0, 0.25, 0.5, and 1.0 mg/ml) at 37 °C for 0.5, 1.0, 2.0, or 4.0 h, respectively. Bacterial pellets were lysed using 400 μl of buffer (150 mM NaCl, 1% Triton X-100, 1 × protease inhibitor, and 50 mM HEPES-Na, pH 8.0) with sonication and freeze-thaw cycles. Supernatants were extracted with methanol/acetonitrile and analysed by LC-MS/MS (Agilent 1260 HPLC-AB SCIEX QTRAP 6500) using a HSS T3 column (2.1 × 100 mm, 3.0 μm) with mobile phase A (20 mM ammonium formate in water) and mobile phase B (methanol). The flow rate was set to 0.3 ml/min. The linear gradient was as follows: 0.1–1.0 min, 98% A; 1.0–5.0 min, 98%–10% A; 5.0–6.0 min, 10%–0% A. Considering the rapid metabolism of TP into T *in vivo*,^25^ the intracellular level of T was also included in the total intracellular content of TP-derived compounds (TP + T). Both TP (*m/z* 451, negative mode)^25^ and its metabolite T (*m/z* 371, positive mode)^25^ were quantified using multiple reaction monitoring (MRM).

### Enzyme activity of ALP

ALP activity was analysed using a modified protocol.^27^^,^^28^ Briefly, mid-log-phase *MRSA* or *MRSE* were treated with lysozyme, TP, or their combination at different concentrations for 1 h at 37 °C. Those without any treatment served as control group. Bacterial suspensions (5.0 × 10^12^ CFU/ml) were sonicated (40 min, 4.0 °C) and centrifuged (18,000 *g*, 20 min, 4.0 °C). Next, 20 μl of each supernatant was incubated with 4.0 μg TP (1 h at 37 °C) and analysed by HPLC-MS (Agilent 1260-AB SCIEX QTRAP 6500). For visualisation, bacterial suspensions were exposed to BCIP/NBT working solution (Beyotime Biotechnology, China) for 30 min at 37 °C in the absence of light, fixed in 3.7% formaldehyde, and examined by fluorescence microscopy (×1500) (representative images were shown). Quantitative analysis used lysate supernatants with absorption spectra (300–800 nm) measurement.

### Skin infection assay

According to the reported method,^9^ 15 Sprague Dawley (SD) rats (6–7 weeks old, from Charles River Laboratories, Inc.) were divided into 5 groups (3 rats per group). Among them, 12 rats were infected with 5.0 × 10^11^ CFUs of mid-log phase *MRSA* applied to 3.0 cm × 3.0 cm dorsal wounds after depilation and anaesthesia. Bacterial suspensions were prepared by centrifugation and resuspension in PBS to 4.0 × 10^11^ CFU/ml.^9^ Uninfected rats received saline instead of bacteria. After 3 h infection, wounds were covered with saline-soaked sterile gauze (0.2 g/cm^2^). After 24 h of infection, these infected wounds received treatments of: saline (control), TP (0.167 mg/kg), lysozyme (0.833 mg/kg), or a combination of both (0.167 mg/kg of TP and 0.833 mg/kg of lysozyme). Meanwhile, rats in the uninfected group were treated with new sterile gauzes soaked in normal saline. This procedure was repeated at 48, 72, 96, 120, and 144 h post-inoculation. At 168 h post-inoculation, all rats were sacrificed to collect their wound tissues. Collected wound tissues were either fixed in 10% formalin for paraffin embedding and haematoxylin and eosin (H&E) staining (4.0 μm sections), or homogenised in PBS for measurement of reactive oxygen species (ROS) and interleukin-6 (IL-6), bacterial quantification via serial dilution, and storage at −80 °C for further analysis.

### Ocular infection model and microbiome investigation

According to the reported method,^29^ 40 C57BL/6 mice (6–8 weeks old, 18–22 g, from Charles River Laboratories, Inc.) were divided into five groups (one uninfected and four infected groups, eight mice per group). Mid-log phase *MRSA* was prepared from overnight cultures, washed, and resuspended to 1.0 × 10^10^ CFU/ml in PBS. Mice were anaesthetised and inoculated with 2.5 μl bacterial suspension (2.5 × 10^7^ CFUs) or PBS onto each ocular surface (two surfaces per mouse). At 6-h post-infection, eight infected mice without any treatment served as the control infected group. Meanwhile, mice in the other infected groups received monotherapy with either TP (0.125 mg/ml, 2.5 μl/eye) or lysozyme (100 mg/ml, 2.5 μl/eye), or a combination therapy (0.125 mg/ml TP + 100 mg/ml lysozyme, 2.5 μl/eye in total). Afterwards, all mice were removed from the air-anaesthetising room and monitored until they were fully awake, after which they were returned to their cages. Treatments were repeated at 24-h. Notably, at 24-h and 48-h post-inoculation, tear fluid was collected (prior to the procedure, 5.0 μl of PBS was administered to each ocular surface) for bacterial quantification^29^ and stored at −80 °C for analysis. Corneal damage was assessed at 24-h and 48-h by staining the right eye using 1.0 μl of 1% sodium fluorescein, followed by slit-lamp biomicroscopy under cobalt-blue light to photograph each cornea. The staining of each corneal zone (*i.e.,* superior, inferior, temporal, nasal, and central) was scored as follows: zero (absent), one (regional or diffuse punctate staining and moderate stipple staining), two (heavy stippling and dense coalescent staining), or three (diffuse loss of epithelium).^29^ All mice were sacrificed at 48-h post-inoculation.

To evaluate the combination effect on the host defence against *MRSA* infection, the total protein content in the collected tear fluid and ocular homogenate was first analysed using a standard BCA assay. Next, tear fluids from unstained eyes were centrifuged (250 *g*, 10.0 min, 4.0 °C) to isolate neutrophils/epithelial cells. These cells were re-suspended in 5.0 μl of PBS and mounted on slides using cytospin centrifugation for subsequent staining using Hema-3 kit.^30^ For each slide, one hundred randomly selected epithelial cells per slide were analysed at 400× magnification under light microscopy. The invasion percentage of epithelial cells was defined as the percentage of epithelial cells invaded by one or more bacteria compared to the total number of epithelial cells observed on the slide. The ratio of the number of observed bacteria to the number of epithelial cells served as the average number of bacteria per epithelial cell. Besides, by normalising GAPDH, surfactant D (SP-D) levels in tears and ocular homogenates were analysed using relative antibody (1:1000 dilution, Abcam, China).^30^

For the H&E analysis of the lacrimal glands, upper eyelids with conjunctiva, and eyeballs of mice, the collected tissues were first fixed in 10% formalin for a minimum of 24 h and then embedded in paraffin. 4.0 μm sections from three mice per group were analysed. The xylene-deparaffinized sections were washed in 100% ethanol, rehydrated in graded ethanol, and incubated with H&E staining solution according to the reported method.^30^^,^^31^

To investigate microbiome, tear fluid (from unstained eyes at the 24-h and 48-h) and 100 μl of left ocular homogenate (at 48-h) were collected to conduct 16S rRNA analysis (BGI Genomics, China). The V3 and V4 regions of the bacterial genome were specifically amplified to analyse the composition of different microbes within the microbial community.^32^, ^33^, ^34^

### Ethics and materials

All animal experiments were approved by and conducted in accordance with the guidelines established by the Committee on the Use of Live Animals in Teaching and Research (CULATR) (Ref No.: 21-22/262-ABCT-R-OTHERS) at The Hong Kong Polytechnic University (Shenzhen Research Institute). All compounds and equipment were purchased from MCE (China) and GE Healthcare (USA) unless otherwise stated.

### Statistical analysis

All assays were performed in triplicate, and results are expressed as the mean ± SD unless otherwise stated. Pairwise comparisons between two groups were assessed using two-tailed Student's t-tests (∗*p* < 0.05, ∗∗*p* < 0.01 and ∗∗∗*p* < 0.001). All individual graphs were generated using GraphPad Prism 9.0, with consistent formatting applied through Adobe Illustrator 2023 for unified visual presentation unless otherwise stated. Detailed statistical parameters (including the sample sizes for each experiment) were provided in respective figure legends.

### Role of funders

This project was supported by the following funding sources: Collaborative Research Funds (CRF) from the Research Grants Council (RGC) of Hong Kong (C5033-19E, C7103-22GF), General Research Funds (GRF) from the RGC of Hong Kong (15305821, 15307122), Redbird Innovation Fund (RIF) from the RGC of Hong Kong (R5008-22), Midstream Research Programme for Universities (MRP) under the Innovation and Technology Fund (ITF) of Hong Kong (MRP/043/21), Collaborative Research Scheme (CRS) between the National Natural Science Foundation of China (NSFC) and the RGC of Hong Kong (CRS_HKUST605/22). The funders had no role in the study design, data collection, data analyses, interpretation, or writing of the report.

## Results

### Re-sensitisation was shown in representative combination between lysozyme and tedizolid phosphate

**Fig. 1:**
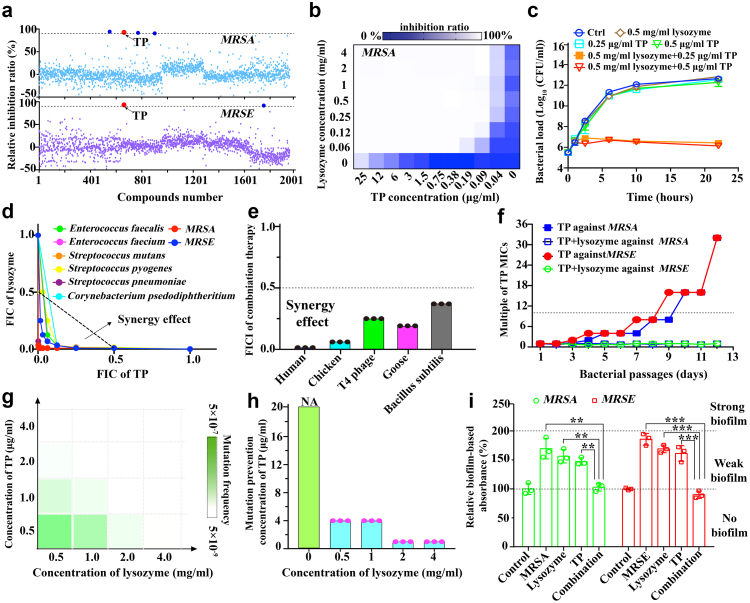
**High-throughput screening revealed a synergistic effect between TP and lysozyme. (a)** The relative inhibition ratios of *MRSA* (upper) and *MRSE* (lower) treated with 1953 FDA-approved drugs in the presence or absence of lysozyme. Cut-off: 90% of relative inhibition ratio. Compared with these blue dots (from left to right: chlorhexidine, tilimicosin, mezlocillin sodium, and nitazoxanide), only red dot (TP) was observed the double inhibition. **(b)** A representative heat-map showing the inhibited growth of *MRSA* under combination therapy. **(c)** Time-kill curves for log-phage *MRSE* under indicated treatments for 22 h. **(d)** Isobolograms of combination therapies against eight different drug-resistant Gram-positive pathogens. **(e)** The FICI analyses on *MRSA* between TP and five sub-types of lysozymes. **(f)** Resistance acquisition curves during 12 days of serial passages in either *MRSA* or *MRSE* treated with the sub-inhibitory concentration of TP or combination therapy with identical concentration of lysozyme. **(g)** Representative heat maps of mutation frequencies of *MRSA* exposed to lysozyme, TP or their combination at different concentrations. It was performed using bacteria continuously cultured on agar plates, which corresponds to the first passage. **(h)** A bar plot showing mutation prevention concentrations of TP in the presence or absence of lysozyme. Bacteria were similarly cultured (first passage). **(i)** Crystal violet assays on *MRSA* and *MRSE* to show the formation of biofilm under indicated treatments. Two-tailed Student's t-tests were applied with the significance thresholds: ∗∗*p* < 0.01 and ∗∗∗*p* < 0.001. All assays were performed in triplicate (three independent replicates per group).

Using the most notorious methicillin-resistant *S. aureus* USA300 (*MRSA*, ATCC-BAA-1556) and *S. epidermidis* (*MRSE*, ATCC-35984) as representative strains, we had an initial thought to cross-evaluate anti-bacterial activities of 1953 FDA-approved drugs in the absence or presence of natural bio-molecules, such as glucose and lysozyme, regardless of whether they worked through known functions or as adjuvants in these combinations, to maximise the safe potential for future applications (Fig. 1a and Figure S1a–c). Among them, tedizolid phosphate (TP), the newest FDA-approved oxazolidinone-class antibiotic, demonstrated synergistic effects with lysozyme against both *MRSA* and *MRSE*, even though two pathogens had been resistant to both TP and lysozyme according to the guidelines of The Clinical & Laboratory Standards Institute (CLSI)^35^ (Fig. 1b, Figure S1d–f and Table S1). In contrast, no synergistic effect was observed when lysozyme was combined with tedizolid (T), the active form of TP in bacterial cytoplasm (Figure S1e). As a prodrug with phosphorylation-modification, TP is widely applied to treat Gram-positive bacterial infections in clinical settings, particularly in cases involving tissues such as the skin, which harbour diverse microbiomes.^6^

To verify this synergistic effect, we then conducted standard checkerboard micro-broth dilution assays and demonstrated that lysozyme at a concentration of *ca*. 0.125 mg/ml (equivalent to 1/8 physiological concentrations, <0.0009 MIC) could synergise with TP to eliminate both *MRSA* and *MRSE*, with a FICI below 0.023 (FICI< 0.5 indicating synergism, Fig. 1b and Figure S1d). This, in turn, resulted in a ≥500-fold reduction in the MIC value of TP, from ≥50 μg/ml to 0.1 μg/ml (Figure S1f). There was the same observation in the time-kill curve,^2^^,^^9^ where bacterial growth was fully inhibited under combination treatment, with bacterial load per ml culture dropping from the levels of 10^12^ to 10^6^ CFUs after 22 h of incubation (Fig. 1c and Figure S1g–i).

Next, we tested the efficacy of this representative combination therapy against various Gram-positive pathogens, including *Enterococcus faecium* (one of the most lethal “*ESKAPE*” pathogens, vancomycin-resistant strain, CGMCC code: 1.15321), *Enterococcus faecalis* (vancomycin-resistant strain, CGMCC code: 1.10682), *Streptococcus pyogenes* (CGMCC code: 1.8868), *Streptococcus mutans* (CGMCC code: 1.2499), *Streptococcus pneumoniae* (CGMCC code: 1.8722), and *Corynebacterium pseudodiphtheriticum* (CGMCC code: 1.592) (Figure S1j). Synergistic effects were successfully observed in all tested groups and their FICI values ranged from 0.015 to 0.156 (Fig. 1d). This phenomenon was also observed when TP was combined with other sub-types of widely-used lysozymes in nature, *i.e.,* C-type (in human and chicken, the most studied), G-type (in goose), T4-type (in T4 bacteriophage) and bacterial-type lysozyme (in bacteria)^17^^,^^21^ (Fig. 1e and Figure S1k). All indicated that this combination strategy had a similar broad-spectrum effect and potential common bactericidal mechanism.

Then, to evaluate the potential risk of generating AMR, bacterial continuous passages were performed. Specifically, after 12 days of serial passages (one passage per day, counted as one generation), we found that the MIC level of TP increased over 16-fold in bacteria exposed to TP alone but remained unchanged under combination therapy (Fig. 1f). This combination therapy not only efficiently reduced the minimal mutation prevention concentrations^2^^,^^9^ of TP from >50 to ≤1.0 μg/ml but also narrowed bacterial mutation frequencies from ≥5 × l0^−7^ to ≤2 × l0^−9^ in both *MRSA* and *MRSE* (Fig. 1g and h, Figure S1l and m). These results indicated that this combination strategy could effectively suppress the development and even minimise *de novo* emergence of drug resistance.

It is known that the presence of biofilm would exacerbate the emergence of AMR.^15^ Therefore, we subsequently performed the crystal-violet assay to evaluate the inhibitory effect of combination strategy against biofilm.^15^ Either TP or lysozyme alone had minimal inhibitory effects on biofilm formation, as indicated from small reduction in absorbance at 590 nm. Remarkably, biofilm was successfully eliminated under combination treatment (Fig. 1i). Similar inhibitory effect was also observed even after biofilm formation, as biofilm-based bacterial growth in fresh sterile LB medium was thoroughly inhibited (Figure S1n). Considering the remarkable efficacy of human lysozyme combined with TP, this combination was established as the representative formulation in subsequent studies.

### Activated alkaline phosphatase under combination therapy enhanced the total levels of intracellular TP-derived compounds

**Fig. 2:**
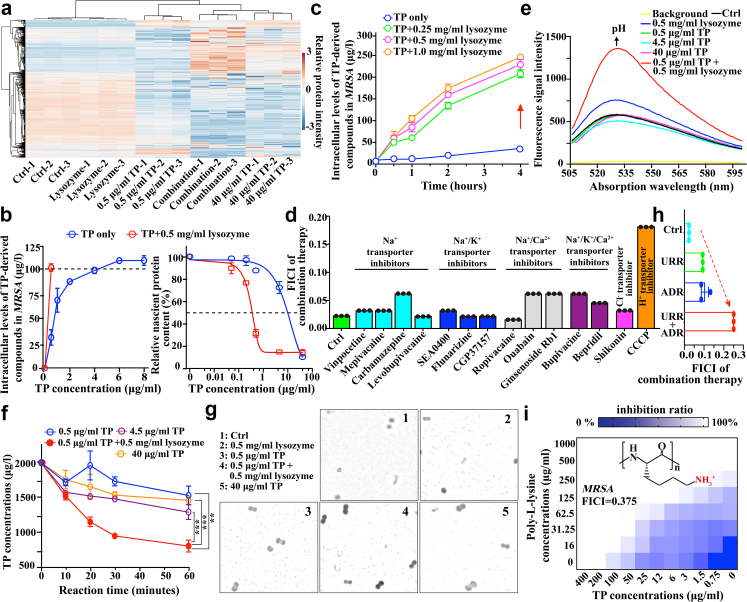
**Combination therapy disrupted the urea cycle, leading to an alkalisation of the cytoplasm and subsequently activating ALP, which prompted the intracellular levels of TP-derived compounds. (a)** A heat map illustrating the DEPs in *MRSA* under different treatments, highlighting the high similarity between the combination group and 40 μg/ml TP group. **(b)** Dose-dependent analyses on intracellular levels of TP-derived compounds (left) and synthesis of nascent proteins (right) in *MRSA* after 1 h treatments. The dotted lines indicate the intracellular level of TP (left) and the half inhibitory concentration (right), respectively. **(c)** Time-dependent analysis on intracellular levels of TP-derived compounds in *MRSA*, showing that combination therapies increased the intracellular TP content. **(d)** FICI analysis of combination therapies against *MRSA* exposed to different ion-targeting inhibitors. The increasing FICI in the CCCP-treatment group indicated that H^+^ may play a role in the combination therapy. **(e)** Fluorescence signal intensities at 520 nm from a pH-sensitive fluorescence probe BCECF-AM increased when *MRSE* was treated with combination therapies for 1 h, indicating a higher alkaline cytoplasmic pH level in the combination group. **(f)** Rate studies on the hydrolysis of TP by ALP extracted from *MRSA* under different treatments *in vitro*. **(g)** Representative micrographs showing the catalytic activity of ALP. The dark pigment represented the product (NBT-formazan) catalysed by ALP. Each dot represented one bacterium. **(h)** FICI analyses of combination therapies against *MRSA*, with or without the exposure to urease inhibitor (abbreviated as URR) and/or arginine deiminase inhibitor (abbreviated as ADR). **(i)** Successful observation of the synergistic effect of combination therapy involving TP and poly-l-lysine, a randomly selected representative compound that exhibits a significant positive charge under physiological conditions. The inset indicates the chemical structure of poly-l-lysine. Two-tailed Student's t-tests were applied with the significance thresholds: ∗∗*p* < 0.01 and ∗∗∗*p* < 0.001. All assays were performed in triplicate (three independent replicates per group).

**Fig. 3:**
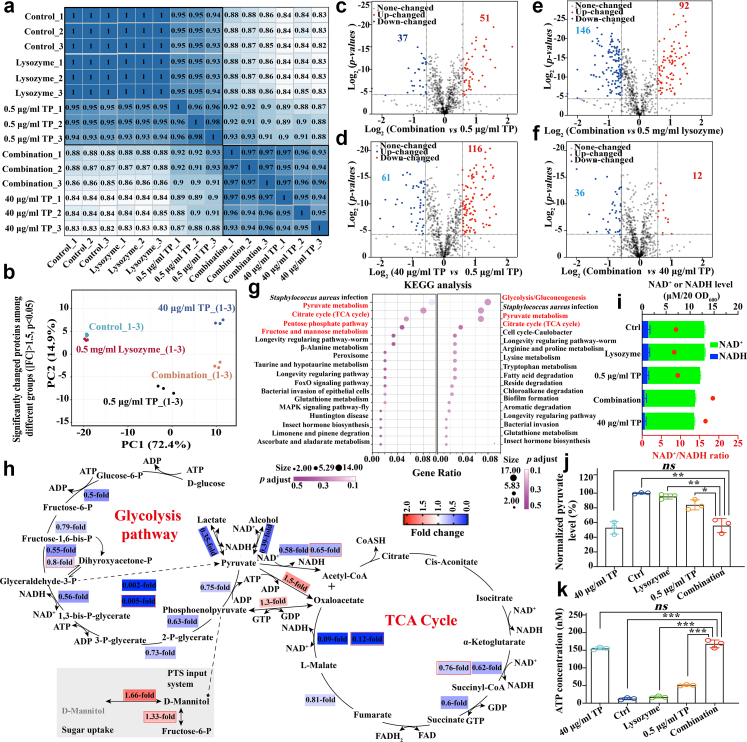
**Proteomic analysis of *MRSA* revealed that the combination therapy modulated energy metabolism and adopted the same mechanism with high dose of TP mono-therapy. (a)** Heat map of correlation coefficients among different groups. The value in each combination-box indicated the relative *Pearson* correlation coefficient. **(b)** Two principal components defined for analysing changes in proteins expression in the proteomics study, showing higher similarities between the combination therapy group and 40 μg/ml TP group were clearly observed. **(c–f)** Volcano plot of proteins in *MRSA* treated with **(c)** combination therapy or 0.5 μg/ml TP; **(d)** 40 μg/ml TP or 0.5 μg/ml TP; **(e)** combination therapy or 0.5 mg/ml lysozyme; and **(f)** combination therapy or 40 μg/ml TP. Total numbers of up-regulated or down-regulated proteins are highlighted in red or cyan in the volcano plot map. **(g)** KEGG analyses of the differential expression proteins in *MRSA* treated with (*left*) combination therapy or (*right*) 40 μg/ml TP if compared with those treated with 0.5 μg/ml TP alone for 1 h. Some critical signal pathways in energy metabolism were highlighted in red areas. **(h)** The diagram illustrates the inhibited glycolysis pathway and TCA cycle, with up-/down-regulated proteins highlighted in red/blue areas. Numbers in the highlighted areas indicate the fold-change of proteins in *MRSA* treated with combination therapy compared to TP alone. **(i–k)** Analyses of the levels of **(i)** NAD^+^/NADH, **(j)** pyruvate, and **(k)** ATP in *MRSA* under 1 h different treatments. These metabolite levels were quantified using commercial assay kits with supernatants from mid-log phase *MRSA* (5.0 × 10^6^ CFU/ml) following 40 min sonication. Untreated bacteria served as controls (intensity set as 100% for normalisation). For NAD^+^/NADH analysis, NAD^+^ was selectively decomposed by heat treatment (60 °C, 30 min) to differentiate NADH alone (heated) and total NAD(H) (unheated). Two-tailed Student's t-tests were applied with a significance threshold: ∗*p* < 0.05, ∗∗*p* < 0.01 and ∗∗∗*p* < 0.001. All assays were performed in triplicate (three independent replicates per group).

To understand which forms of oxazolidinone-class antibiotics and whether their combinations with lysozyme eliminated pathogens through mechanisms different from those of monotherapies, we conducted a TMT-based quantitative proteomic profiling of *MRSA* under different treatments (Table S2). Before that, it was observed that bacteria treated with 0.5 μg/ml TP (designated as the low dose) in the presence of 0.5 mg/ml human lysozyme (hereafter referred to as combination therapy) achieved the comparable OD_600_ to those treated with 40 μg/ml TP (designated as the high dose), which was rarely observed in these groups treated with T (control: no drug treatment; lysozyme monotherapy: 0.5 mg/ml human lysozyme; low dose TP monotherapy: 0.5 μg/ml TP; high dose TP monotherapy: 40 μg/ml TP; combination therapy: 0.5 mg/ml human lysozyme and 0.5 μg/ml TP) (Figure S2a and b). Interestingly, in the heat-maps showing differentially expressed proteins (DEPs), bacteria treated with high-dose TP and combination therapy exhibited very similar profiles (Fig. 2a), with a *Pearson* correlation coefficient R^2^ of 0.95 and a high correlation in the principal component analysis (PCA), indicating similar bactericidal mechanisms between TP alone and combination therapy (Fig. 3a–f). In contrast, lysozyme itself was not able to give such change in proteome in general (Fig. 2a). All indicated that combination therapy might eliminate pathogens through a mechanism similar to that of TP monotherapy.

To evaluate this, we then assessed the extent of nascent protein inhibition in *MRSA* under different treatments using a probe OPP since oxazolidinone-class antibiotics, including TP and its active metabolite T (collectively TP-derived compounds), act by inhibiting bacterial protein synthesis.^4^, ^5^, ^6^ As shown in Fig. 2b, the half inhibitory concentration (IC_50_) of TP to inhibit protein synthesis in combination therapy or monotherapy was calculated as 0.32 or 9.5 μg/ml, respectively. Thus, we hypothesised that combination therapy might increase the total levels of intracellular TP-derived compounds and subsequently lead to enhanced inhibitory effects. To test this hypothesis, we next measured the intracellular levels of TP/T in *MRSA* exposed for various durations or to various concentrations of TP in the absence or presence of lysozyme by using MRM-based MS method.^24^ It was observed that a 4-h combination treatment led to a ten-fold increase in the intracellular levels of TP-derived compounds in *MRSA* (Fig. 2c). Additionally, when *MRSA* was exposed to 0.5 μg/ml TP for 1 h, the intracellular levels of TP-derived compounds quickly reached a concentration of *ca*. 100 μg/l in *MRSA* under combination therapy. Such accumulation could not be achieved for TP monotherapy unless ≥4.5 μg/ml TP was applied (Fig. 2b). Importantly, prolonged incubation time (*e.g.,* 4 h) further enhanced this accumulation, ultimately achieving intracellular levels of TP-derived compounds comparable to those observed with 40 μg/ml TP monotherapy (Figure S3a). This might be attributed to the extension of the saturation dosage in TP monotherapy through sustained exposure, mirroring the pharmacokinetic profile of high-dose monotherapy. Taken together, these findings indicated that lysozyme (as an adjuvant in combination therapy) increased intracellular levels of TP-derived compounds and thus resulted in a stronger bactericidal effect.

To determine whether the increase in intracellular levels of TP-derived compounds was due to damage to cell membrane or cell wall in *MRSA* under combination treatment, we conducted permeability assays using propidium iodide (PI), a red-fluorescent DNA dye that crosses plasma membrane of nonviable cells.^24^ We found no difference in PI-based fluorescence signals, indicating no damage to the cell membrane (Figure S2c). Cell wall integrity was also maintained regardless of how treatments were used, as evidenced by unchanged signals from fluorochrome-SYBR-gold, a diffusible nucleic acid dye that only penetrates when the cell wall is damaged^36^ (Figure S2d). The conclusion was further supported by the observation that when *MRSA* was pre-treated with lysozyme to remove cell walls (if it can) and washed, subsequent TP treatment failed to increase intracellular level of TP-derived compounds and kill bacteria (Figure S3a and b). Considering the critical roles of efflux pumps or transporters in antibiotic resistance,^37^, ^38^, ^39^ we further investigated their potential effects on combination therapy. No change in the FICI of combination therapy was observed when *MRSA* was exposed to 20 inhibitors targeting some well-known efflux pumps^38^ (Figure S2e and f). Interestingly, carbonyl cyanide *m*-chlorophenyl hydrazine (CCCP),^2^ an H^+^ transporter inhibitor, changed the FICI of combination therapy, which was distinct from other ions-targeting transporter inhibitors (Fig. 2d and Figure S2g). Such result indicated that H^+^ played a critical role in synergising TP with lysozyme. This conclusion was further strengthened by membrane potential analyses, where combination therapy reduced the membrane potential, as indicated by an increased fluorescence ratio (green/red) (Figure S2h). Considering the significance of proton/H^+^ in pH homoeostasis,^40^ the intracellular pH level was further determined by using the pH-sensitive fluorescence probe BCECF-AM.^24^ As expected, the combination alkalised bacterial cytoplasm, which was consistent with the variations in synergistic effects caused by lysozyme from different sources, further supporting prior conclusions that pH homoeostasis was disrupted under combination therapy (Fig. 2e, Figures S2i and S3c).

**Fig. 4:**
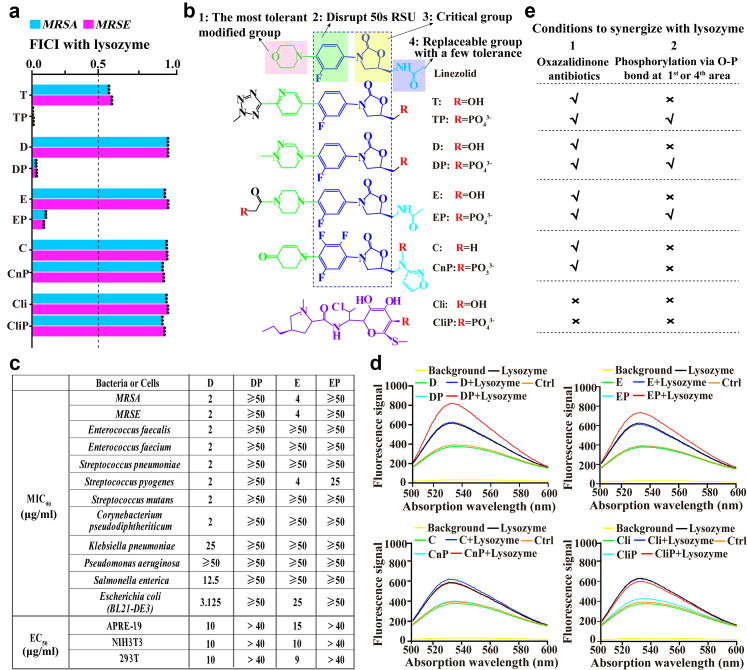
**A strategy to achieve broad-spectrum synergistic effects between various oxazolidinone-class antibiotics and lysozyme. (a)** Comparison of FICIs for several combination therapies, in which lysozyme was used as representative adjuvant in combination with tedizolid (T), tedizolid phosphate (TP), delpazolid (D), delpazolid phosphate (DP), eperezolid (E), eperezolid phosphate (EP), contezolid (C), contezolid phosphoramidic acid (CnP), clindamycin (Cli), or clindamycin phosphate (CliP). Synergistic effects were defined as FICI < 0.5 (dash line). **(b)** Structural comparisons among tested oxazolidinone-class antibiotics (upper five) and lincosamide antibiotics (lower one). Here, linezolid was demonstrated as an example of oxazolidinone-class antibiotics to show the four areas of oxazolidinone-class antibiotics, in which the core structures of oxazolidinone-class antibiotics (2nd area: to disrupt bacterial 50S ribosomal subunit; 3rd area: critical group due to H-bond and conformational effects) were highlighted in the blue box. Both 1st area and 4th area were the tolerant modified groups in structural modification. **(c)** Antibacterial activities (MIC_90_) and bio-toxicities (median effect concentration, EC_50_) of several selected antibiotics and their synthesised prodrugs. **(d)** The BCECF-AM-based fluorescence signal in *MRSA* under indicated treatments for 1 h. Compared with others, only DP and EP were observed the observable increase in intensities at 520 nm if bacteria were treated in the presence of lysozyme. **(e)** The diagram to summary two conditions if oxazolidinone-class antibiotics were expected to synergise with lysozyme, *i.e.,* oxazolidinone-class antibiotics and phosphorylation on hydroxyl groups via O-P bonds. All assays were performed in triplicate (three independent replicates per group).

Considering that TP is hydrolysed into T by endogenous ALP instead of lysozyme (Figure S3d),^5^ we wondered whether ALP would be activated, and then accelerated the hydrolysis metabolism of TP under the alkalised cytoplasmic micro-environment. To test it, by using the MRM-based MS method,^24^ we analysed *in vitro* hydrolysis rate of TP and observed that up to 65% of TP was hydrolysed in the presence of lysozyme after 1 h, which was significantly faster than other treatments (less than 32%) (Fig. 2f). To directly visualise the *in cellular* activity of ALP, we subsequently analysed bacterial microscopy images, in which 5-bromo-4-chloro-3-indolyl phosphate (BCIP) was added and hydrolysed by ALP, followed by a reaction with nitro blue tetrazolium (NBT) to become insoluble brown products (NBT-formazan, with maximal absorbance at 530 nm). As shown in Fig. 2g, the combination treatment resulted in more prominent brown-black dots (each dot representing one bacterium). Consistent with this, bacterial supernatant in the combination group was also observed a greater absorbance intensity at 530 nm (Figure S2j), indicating that bacterial ALP was activated under combination therapy. However, when the activation was blocked by ALP inhibitor levamisole, both the synergistic effect and the characteristic intracellular accumulation of TP-derived compounds were abolished (Figure S3a and e). Similar phenomena were observed when FCCP (carbonyl cyanide 4-(trifluoromethoxy)phenylhydrazone), which acidifies the cytoplasm, was added (Figure S3a). This confirmed that the alkaline pH-activated ALP hydrolysis directly mediated the increase in TP-derived compound levels, as unmetabolized TP would otherwise accumulate. Together, the activation would strengthen the metabolic sink that drives further net TP influx to maintain a balance for subsequent consumption in TP-to-T conversion. To further confirm that the ALP-based hydrolysis to remove phosphate group was crucial in the synergistic effect, we next compared the FICIs of combination therapies if lysozyme was combined with TP's analogues without phosphorylation modification, *i.e.,* T or linezolid.^4^, ^5^, ^6^ No synergistic effect was found in these combinations (Fig. 4a and Figure S2b). These findings indicated that combination strategy alkalised cytoplasmic pH level, thereby activating ALP and further underscoring the significance of the phosphate group in increasing the intracellular levels of TP-derived compounds in combination with lysozyme.

### To outline a novel class of re-sensitisation strategies for diverse oxazolidinone-class antibiotics

Based on this principle, we next introduced a practical strategy to explore whether oxazolidinone-class antibiotics with specific characteristics could be also synergised in combination with lysozyme (as the representative pH homoeostasis-breaker). Considering that prodrugs with phosphorylation modification are widely documented in the pharmaceutical industry,^7^^,^^8^ we next selected another two representative oxazolidinone-class antibiotics, *i.e.,* delpazolid (D) and eperezolid (E), and chemically synthesised their phosphorylated analogues/prodrugs via hydroxyl groups located on both sides of their core structures (Fig. 4b and c, Figure S4a–c). Excitingly, both delpazolid phosphate (DP) and eperezolid phosphate (EP) exhibited strong synergistic effects in combination with lysozyme (FICI ≤ 0.125, Fig. 4a and Figure S4d). In contrast, another oxazolidinone-class antibiotic named contezolid phosphoramidic acid (CnP), which underwent phosphorylation for nitrogen instead of oxygen, did not show any synergy with lysozyme (Fig. 4a and b, Figure S4d). This difference is reasonable because this modification of nitrogen, although it is frequently reported, resulted in a slower drug-conversion rate in ALP-based hydrolysis.^41^ Meanwhile, clindamycin phosphate (CliP), a lincosamide-class rather than oxazolidinone-class antibiotic, exhibited minimal synergy with lysozyme, although its hydroxyl group was phosphorylated (Fig. 4a and b). This suggests that oxazolidinone-class antibiotics themselves may also play an irreplaceable role in the combination with lysozyme. Additionally, to further confirm whether the cytoplasmic micro-environment was also alkalised in bacteria treated with DP or EP in combination with lysozyme, we performed BCECF-AM-based probe assays on these bacteria. As shown in Fig. 4d, both DP and EP resulted in the notable increase in intensities at 520 nm if bacteria were co-treated with lysozyme, indicating that pH homoeostasis did been disrupted. In contrast, such changes did not occur for other treatments.

In summary, such synergetic effect in combination with lysozyme occurred for these oxazolidinone-class antibiotics with specific characters (Fig. 4e). Specifically, we developed a novel class of re-sensitisation strategies for various oxazolidinone-class antibiotics, which require these oxazolidinone-class antibiotics to be phosphorylated via O-P bonds (the practical path of prodrug development), regardless of which sub-types of lysozymes are combined because the ability of lysozyme as pH homoeostasis-breaker in combination therapy is independent of its well-known antibacterial role (glycan hydrolase activity).

### Combination disrupted urea cycle and alkalised cytoplasm, in which lysozyme stood out as adjuvant

**Fig. 5:**
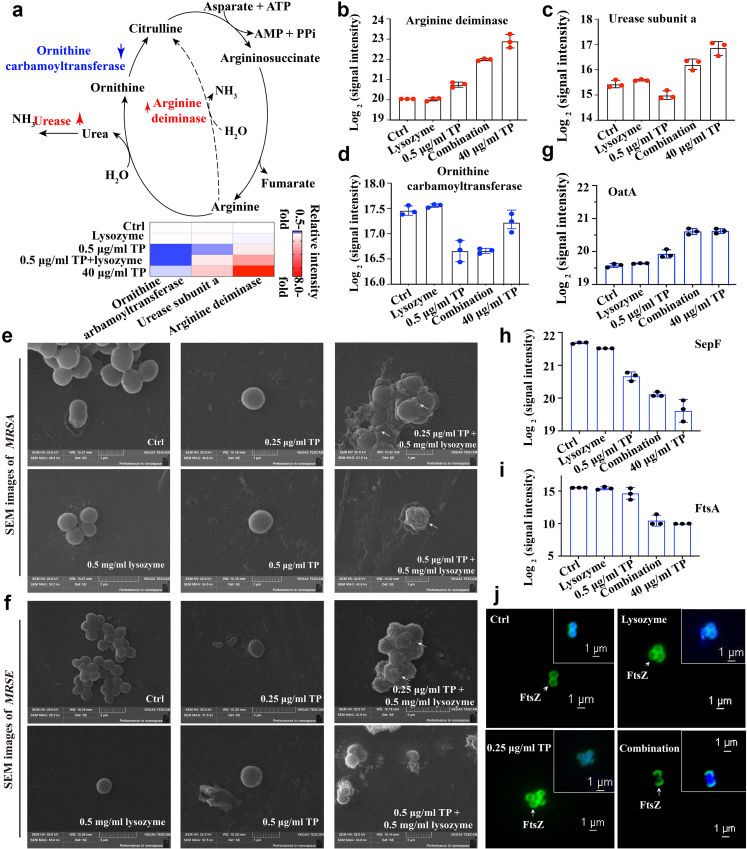
**Disrupted urea cycle, cell division and change in bacterial morphology. (a)** Diagram illustrating urea cycle, in which up-/down-regulated proteins highlighted in red/blue areas. Fold-changes of **(b)** arginine deiminase, **(c)** urease subunit alpha, and **(d)** ornithine carbamoyltransferase. **(e–f)** SEM of **(e)***MRSA* and **(f)***MRSE* under different treatments. Numerous cytolysis sites and bud-like humps (white arrowheads) were observed in the combination group. **(g–i)** Fold-change of three critical cell division enzymes, *i.e.,***(g)** OatA, **(h)** SepF, and **(i)** FtsA in proteomics studies. **(j)** FtsZ-based immunofluorescence images on *MRSA* under different treatments, in which combination therapy led to “crescent-shaped” Z-ring and uneven cell division. Log-phase *MRSA* was treated with 0.25 μg/ml TP, 0.5 mg/ml lysozyme, or their combination for 1 h at 37 °C. After fixation and permeabilization, bacteria were enzymatically treated (25 μg/ml of lysozyme and 50 U/ml of DNase I) and blocked. FtsZ was stained using its primary and FITC-conjugated secondary antibodies. Then, samples were mixed with agarose, mounted, and imaged by fluorescence microscopy (representative images shown). All assays were performed in triplicate (three independent replicates per group).

To further investigate the molecular mechanism behind the alkalisation of the cytoplasm in representative combination, we re-analysed the aforementioned proteomic change and observed significant (FDR-adjusted *p* < 0.05) up-regulation of 51 DEPs and down-regulation of 37 DEPs in *MRSA* under combination therapy compared to those treated with 0.5 μg/ml TP alone (Fig. 3c–f). Further analysis revealed that this combination led to the increase in levels of arginine deiminase (3.9-fold) and urease subunit alpha (1.5-fold), as well as the decrease in the level of ornithine carbamoyltransferase (0.5-fold) (Fig. 5a–d). These enzymes play a critical role in the urea cycle.^8^ Such changes in this pathway and the endpoints might cause over-production of urea and ammonia, leading to an alkalised cytoplasmic micro-environment. To test it, we then performed FICI analyses on *MRSA* under combination therapies in the absence and presence of inhibitors targeting arginine deiminase^42^ or urease.^43^ As shown in Fig. 2h, the presence of inhibitors increased FICI from 0.015 to 0.1 (up to 0.25 in the presence of two inhibitors) (Figure S2k–m). A similar conclusion was drawn from a parallel BCECF-AM-based pH-sensitive fluorescence assay (Figure S2n).

Notably, the cellular acid-base regulatory system has a limited ability to overcome dramatic changes in pH, possessing strong capability around neutral pH but considerably weaker ability at pH levels far from homoeostatic pH.^44^^,^^45^ As a basic-residue rich enzyme,^17^ lysozyme is strongly positively charged in the physiological environment, making pH homoeostasis more frangible when cells were exposed to micro-environment containing abundant lysozyme. This may explain why pH homoeostasis was more easily disrupted in bacteria under the combination treatment than TP alone.

Based on this hypothesis, we further analysed the FICI of combination therapy between TP and natural cell adhesion molecule poly-l-lysine (a randomly-selected positively charged compound in physiological condition, Fig. 2i).^46^ It was observed that this compound could successfully reproduce the expected synergistic effect in combination therapy, although the FICI was slightly higher (FICI = 0.375) (Fig. 2i). These proteomic, inhibitor-based and analogue-based evidence supported our proposed mechanism involved in the urea cycle, and extended the adjuvant to a broader range of agents with abundant positive charges.

In summary, compared to small molecular analogues (like poly-l-lysine) or macro-molecular metabolites (like lactoferrin in Figure S1c), lysozyme exhibited the greatest potential as an adjuvant for oxazolidinone-class antibiotics including TP, DP, and EP, as indicated by the lowest FICI values. Such advantage is reasonable because positively charged lysozyme is a bacterial-targeting macro-molecular metabolite with more stable metabolic concentration and longer-lasting effectiveness in applied regions than others, leading to the sustained inhibition of bacterial growth. More importantly, as a natural enzyme metabolite that has demonstrated considerable safety *in vivo*, lysozyme is so well-positioned that it stands out to be combined with various oxazolidinone-class antibiotics, particularly TP, which endows such combination therapy as the representative example in the proposed strategy. Remarkably, as both agents have excellent safety profiles, such clinical investigation may immediately be contemplated in humans.

### Multi-techniques revealed that both bacterial energy metabolism and cell division were unexpectedly disrupted

Considering the potential role of the urea cycle in re-shaping bacterium,^47^ we further conducted the Scanning electron microscopy (SEM) imaging of *MRSA* or *MRSE* under combination therapy to investigate morphological alterations. As shown in Fig. 5e and f, the digestion-like cell lysis was clearly observed in pathogens under combination therapies, indicating bacterial near-death situations in combination groups. Interestingly, we unexpectedly noticed numerous bud-like protuberances in these groups. An in-depth analysis of DEPs revealed the substantial increase in the level of OatA (an O-acetyl transferase promoting bacterial resistance to lysozyme^20^) (Fig. 5g). This overexpression might result from the stress response to lysozyme-exposure. Notably, it was reported that increased OatA would cause abnormal cell division.^20^ Meanwhile, we also observed reductions in SepF (0.5-fold) and FtsA (0.003-fold) levels (Fig. 5h and i). Like OatA, SepF and FtsA play critical roles in cell division including FtsZ bundling, Z-ring tethering and membrane re-shaping activities. Down-regulation of anchors including SepF may cause an uneven Z-ring assembly at the cell division site.^48^^,^^49^ Consequently, alterations in OatA, FtsA and SepF might contribute to defective Z-ring assembly at uneven cell division sites, resulting in the emergence of small nascent bacteria as bud-like protuberances and subsequent bacterial growth inhibition. To test it, we performed an immunofluorescence microscopy assay on *MRSA* treated with an FtsZ antibody targeting Z-rings. As shown in Fig. 5j, lasso-like Z-rings were observed at uneven bud-like junctions (crescent shape, combination group) compared to equal division sites (circle, other groups). This faulty cell division reveals the molecular basis behind these SEM images and indicates an unreported mechanism by which TP inhibits the growth of pathogens.

Meanwhile, gene ontology (GO) annotation analyses on aforementioned DEPs revealed that they were mainly associated with large ribosomal subunit/cytosolic large ribosomal subunit (cellular components), RNA binding/protein-containing complex binding (molecular functions) and organelle organisation/cellular component organisation (biological processes) (Table S2). Kyoto Encyclopedia of Genes and Genomes (KEGG) enrichment analysis demonstrated that these up-/down-regulated DEPs were enriched in the energy metabolism, *i.e.,* pyruvate metabolism, citrate (TCA) cycle, pentose phosphate pathway and fructose/mannose metabolism, indicating that bacterial energy metabolism might be disrupted under combination therapy (Fig. 3g). The similar change was also found in other comparisons (Fig. 3d–g and Table S2). Subsequently, we further observed that various crucial proteins involving in the TCA cycle and glycolysis pathway, such as malate: quinone oxidoreductase 1 (0.12-fold) and phosphorglycerate kinase (0.005-fold) were significantly down-regulated in *MRSA* under combination therapy if compared to that treated with TP alone (Fig. 3h and Table S2). To validate this finding, we next examined intracellular levels of three representative indices of energy metabolism, *i.e.,* NAD^+^/NADH, pyruvate and ATP.^50^ It was observed that combination therapy resulted in an increase in the ratio of NAD^+^/NADH from 8.84 ± 1.89 to 18.43 ± 4.42 (a level comparable to that in *MRSA* treated with 40 μg/ml TP), while both NAD^+^ and NADH levels decreased (Fig. 3i). A similar change was observed in the level of pyruvate (<0.6-fold, Fig. 3j). Notably, unlike two-above indices, the intracellular level of ATP increased (Fig. 3k), which could be also observed in 40 μg/ml TP group. The change might attribute to the saved ATP in the inhibition of nascent protein synthesis (Table S2), as bacterial protein synthesis constitutes a major proportion of energy expenditure.^51^

Together, these findings suggest that beyond the primary mechanism previously described, combination therapy may eliminate pathogens through defective cell division and disrupted energy metabolism as two additional auxiliary pathways. These pathways likely act synergistically, providing a multifaceted explanation for the potent antibacterial synergy observed under combination therapy.

### The in-vivo therapeutic effect of combination therapy

Considering the excellent synergistic effect between TP and lysozyme, along with their established safety profiles, their combination therapy is then conducted as a representative model to evaluate the application potential of combination strategy *in vivo*. We first performed safety analyses on ARPE-19 (retinal pigment epithelial), NIH/3T3 (embryo fibroblast), and 293T (kidney epithelial-like) cells, and found that all three cell lines were insensitive to both TP and lysozyme (Figure S5a–f). Given the abundance of lysozyme in tears, we subsequently selected ARPE-19 cells to evaluate the combination effect on infected cells. In the representative assay, it was observed that a 24-h *MRSA*-infection led to the death of 50% of ARPE-19 cells, which was not rescued by TP or lysozyme monotherapy. However, cell death was prevented when it was treated with ≥1.0 μg/ml TP in the presence of 0.5 mg/ml lysozyme (Figure S5g). Bacterial load also decreased from the levels of 10^7^ to 10^4^ CFU/ml in these infected cells under combination therapy (Figure S5h).

**Fig. 6:**
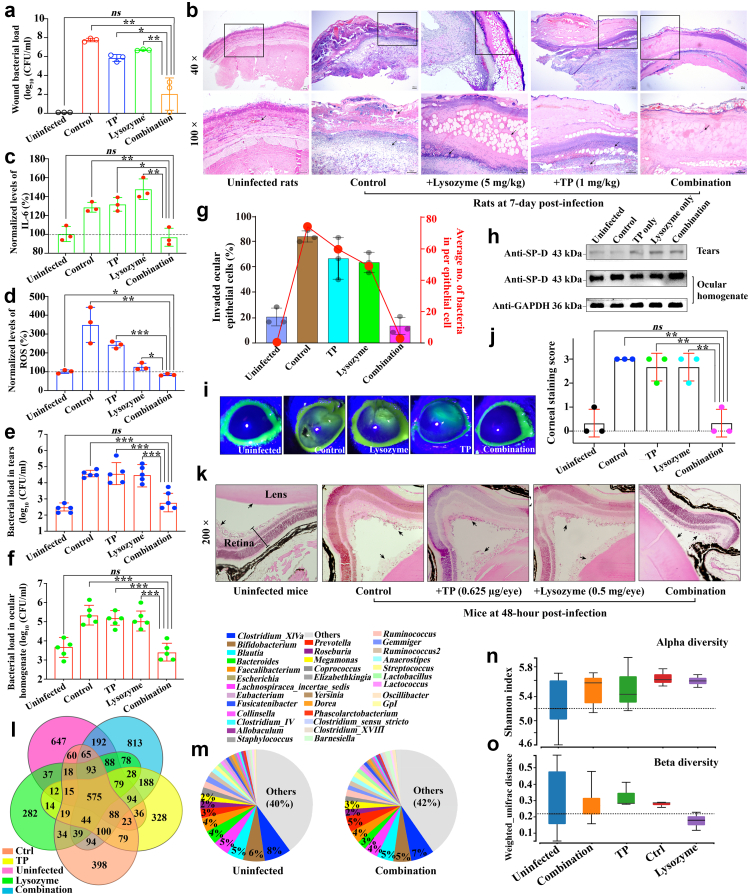
**Combination therapy effectively relieved symptoms in both skin and ocular animal models. (a)** Bacterial load in wounds of infected rats under different treatments for seven days. **(b)** Representative H&E staining images of rat wounds. Two magnifications (upper: 40× and lower: 100×) were available. The combination therapy group showed less dense inflammatory cells with blue nuclei (black arrowheads) and smoother edges. **(c–d)** The normalised levels of **(c)** IL-6 or **(d)** ROS in wounds of rats under different treatments for seven days. **(e–f)** Bacterial loads in **(e)** tear fluids and **(f)** ocular homogenates of C57BL/6 mice under different treatments in the ocular infection model. **(g)** Percentages of invaded epithelial cells in tear fluids of mice under different treatments (left vertical axis), where invaded epithelial cells are defined as those containing one or more bacteria in the Hema-3 staining image (400× magnification). Red dots in the inserts (right vertical axis) show the average number of bacteria per epithelial cell, indicating minimal effects on bacterial infectivity under combination therapy when compared to the uninfected group. **(h)** Normalised expression levels of SP-D in tear fluids and ocular homogenates. GAPDH was used to normalise SP-D levels in ocular homogenates. **(i)** Representative fluorescein images displaying stained corneas of mice after 48-h treatments (left) and **(j)** their eye injury scores (right). Here, 2.5 μl of 1% fluorescein sodium were dropped into each eye to analyse injury according to the ophthalmology scoring standard. **(k)** Representative H&E staining images of eyeballs from C57BL/6 mice under different treatments (200× magnification). Black arrowheads point to infiltrating inflammatory cells and lens edges. Notably, “irregular” ganglion cells were observed in the ganglionic layer of retina. Compared to the combination therapy group, other infected groups exhibited stronger inflammatory response and irregular retinas. **(l)** Venn diagram displaying the detected OTUs in tears of mice under different treatments. 575 core OTUs were shared among different groups. **(m)** Analyses of bacterial community composition in tears from healthy uninfected mice (left) and infected mice under combination therapy (right). **(n)** Alpha and **(o)** beta diversity analyses on various bacterial communities. Whisker lines on each diversity boxplot represent minimum, median and maximum values. Shannon indices and weighted unifrac distances are positively correlated to diversities within one group and among groups. Two-tailed Student's t-tests were applied with the significance thresholds: ∗*p* < 0.05, ∗∗*p* < 0.01, and ∗∗∗*p* < 0.001. Assays were conducted with either three, five, or eight repetitions (three, five, or eight independent replicates per group).

As TP is an FDA-approved oxazolidinone-class antibiotic for treating acute bacterial skin and skin structure infections,^6^ we subsequently evaluated the relieved effect in a rat skin infection model.^9^ We observed a significant reduction in bacterial loads (>6 log_10_) at 168 h post-inoculation when infected rats were treated with combination therapy (1.0 mg TP and 5.0 mg lysozyme/kg rat) (Fig. 6a and Figure S5i). H&E staining showed extensive denser inflammatory cells with blue nuclei in these infected rats that received no treatment or monotherapy with either TP or lysozyme, but not in those under combination therapy, indicating effective alleviation of inflammation (Fig. 6b). The recovery was further confirmed by the decrease in two inflammatory indices, *i.e.,* IL-6 and ROS,^30^ by over 20% and 70%, respectively (Fig. 6c and d). These findings demonstrated the successful translation of combination therapy to *in vivo* treatment.

Considering that lysozyme is a self-secreted macro-molecular cellular metabolite in tears, we next assessed the potential of combination therapy using an ocular infection model. We collected tear fluid and homogenised eye tissues from C57BL/6 mice under different treatments (Figure S6a and b) and observed that combination therapy (0.5 mg lysozyme and 0.625 μg TP/eye) significantly reduced bacterial loads in both tear fluids and ocular homogenates (<−2 log_10_), while monotherapy produced little change (Fig. 6e and f). It was also found that combination therapy proportionally reduced the proportion of invaded epithelial cells and the average bacterial count per epithelial cell in tear fluids, resulting in levels comparable to those in uninfected mice, indicating a negligible risk of TP or lysozyme in enhancing pathogenic infectivity^30^ (Fig. 6g and Figure S6c). Furthermore, we observed that the levels of SP-D (an index reflecting host defence^30^) in tear fluids increased from 0.61-fold to 1.55-fold when infected mice were treated with combination therapy (Fig. 6h). The change in SP-D levels was not observable in ocular homogenates, which could be due to the low proportion of SP-D secreted into tear fluids for protecting the eye from infection.^30^ These findings suggested that combination therapy enhanced the host's innate immune defence against ocular infections. Additionally, greater corneal integrity was also observed in infected mice under combination therapy, as evidenced by reduced ocular fluorescein staining (Fig. 6i and j, Figure S6d and e). Histological analysis of eye tissues revealed that combination therapy led to better recovery, as judged by the smoother edge in the dehydrated vitreous body and more uniform ganglion cells arrangement but less infiltrative inflammatory cells if compared to *MRSA*-infected eye tissues in control or monotherapy groups (Fig. 6k and Figure S6f). Overall, our results indicated that such combination therapy was also highly effective in the treatment of ocular infections.

### The healthy ocular microbiome was successfully restored

More recently, the existence of a microbiome in healthy eyes has challenged the belief that the eye has an almost sterile micro-environment.^52^ Due to a risk of traditional antibiotic (combination) therapy to damage healthy microbiome during the sterilisation of pathogens, we subsequently firstly introduced a concept of healthy microbiome as the evaluated index in antibiotic therapy. We firstly mapped the bacterial communities of the eyeball (ocular homogenate) and ocular surface (tear fluid) of healthy C57BL/6 mice using 16S rRNA meta-barcoding (Figure S7a and b, Figure S8a and b).^53^^,^^54^ To our knowledge, this is the first study to fully analyse the ocular microbiome in these healthy/infected tissues.^13^^,^^55^ A total of 2122 operational taxonomic units (OTUs, reflecting the diversity of bacterial communities) were identified in the tear fluid of healthy mice, of which 575 were shared as core OTUs with other treatments (Fig. 6l). Bacteria in community (cutoff ≥3% proportion, at the genus level) in healthy murine tears were predominantly anaerobic probiotics, including *Clostridium_XlVa* (8%), *Bifidobacterium* (6%), *Blautia* (5%), *Lachnospiracea_incertae_sedis* (5%), *Faecalibacterium* (4%), *Bacteroides* (4%), and *Prevotella* (3%) (Fig. 6m and Figure S7c). It was observed that the proportion of *Staphylococcus* increased from 0.052% (healthy mice) into ∼3% in infected mice but reduced to almost pre-infection levels (∼0.078%) when mice were treated with combination therapy (Table S3), which was consistent with previous CFUs-based evidence. Indeed, according to heat maps and partial least squares-discriminant analysis, it was convincing that the overall bacterial communities were successfully recovered although the proportion of some anaerobic probiotics including *Anaerostipes*, *Propionibacterium*, and *Prevotella* increased^56^ (Figure S7d–g). Additionally, except infected mice under lysozyme monotherapy, greater alpha and beta community diversities were observed in other infected mice than uninfected mice, as indicated by higher Shannon indices and weighted unifrac distances (Fig. 6n and o). According to Graphlan map, bacteria in tears were concentrated in *Firmicutes* and *Bacteriodetes genera* (Figure S7h). Similar trends were also observed in the bacterial communities of ocular homogenates although the degree differed (Figure S8c–k). However, the bacterial community was complex, making it difficult to determine how or whether the growth of bacteria in the network was absolutely inhibited or promoted due to inter-exclusion and co-existence among them (Figures S7i and S8l). In conclusion, these results indicated that such combination therapy between lysozyme and representative oxazolidinone-class antibiotic (TP) was safe and cost-effective because it not only eliminated pathogens from infected mice, but also facilitated the rapid recovery of healthy ocular bacterial communities.

## Discussion

In this study, we developed a practical, translatable and microbiome-friendly strategy to sensitise various oxazolidinone-class antibiotics (TP, DP, and EP). In a representative model, the safe and cost-effective cellular macro-molecular metabolite lysozyme was combined as representative adjuvant against drug-resistant Gram-positive pathogens. This strategy required these oxazolidinone-class antibiotics to be phosphorylated via O-P bonds, in which lysozyme's ability as pH homoeostasis-breaker was independent of its well-known antimicrobial role (glycan hydrolase activity). In this representative combination, the bactericidal activity of TP was enhanced over 500-fold. Specifically, we observed that lysozyme dramatically synergised with TP by disrupting pH homoeostasis and thus activating ALP to accelerate the intracellular accumulation of antibiotic. Proteomic studies on *MRSA* provided additional detailed mechanism. A notable example was the effect on bacterial cell division. In bacteria, cell division occurs through the ingrowth of envelope layers, including membrane and peptidoglycan in the cell wall, which forms a septum to divide the cell into two compartments.^48^ This process is orchestrated by the tubulin homologue FtsZ, which forms a ring-like structure called the Z-ring.^48^^,^^49^ Meanwhile, other essential proteins, such as SepF and FtsA in *MRSA*, are recruited to the division site and activated cell wall synthesis, driving cell envelope constriction.^48^^,^^49^ If these accessory proteins are deficient, the division process may be impaired, leading to undivided compartments.^48^ Other critical factors, such as tightly regulated OatA,^20^ are also crucial for proper cell division. Without them, bacteria may struggle to survive even if cells are successfully but unevenly separated. Our results on TP clearly demonstrated that such combination strategy significantly altered the expression levels of OatA, SepF, and FtsA, leading to uneven and unsuccessful cell division. These effects on cell division regulation represent an unreported antibacterial mechanism of TP, although further studies are needed to confirm this.

This study also assessed the translational potential of combination strategy in skin and ocular infection models (both were mucosal indications) by combining TP with lysozyme as the representative combination. It was observed that combination therapy would lead to a rapid recovery of infected wound and ocular surface. More importantly, we first introduced a conceptual index of whether the healthy microbiome was restored to evaluate the effectiveness of antibiotic treatments in infected tissues and conducted an ocular microbiome-based analysis. To our knowledge, this is the first study to completely analyse the healthy/infected murine ocular microbiome for both the eyeball (ocular homogenate) and ocular surface (tear fluid).^13^^,^^55^ The analysis revealed the diverse bacterial community consisting of several primary anaerobic genera. Indeed, while our *in vitro* assessments focused on aerobic conditions due to the selected pathogens' growth requirements and technical constraints, the ocular infection model revealed that the combination therapy also facilitated the recovery of anaerobic genera (*e.g., Anaerostipes*) in tear fluid. Whether this effect stems from direct action on anaerobes or indirect mechanisms requires further investigation. Despite this, it is still encouraging that this combination facilitates the rapid regeneration of a healthy ocular microbiome, indicating its potential for future clinical application.

Translationally, our strategy enables pharmaceutical scientists to overcome traditional toughie in making trade-offs between efficacy and safety. When antimicrobials (such as T) exhibit low selectivity in targeting pathogens and demonstrate potential toxicities against host cells, their safety can be enhanced by commercialising prodrugs, especially phosphorylated forms (such as TP)^4^^,^^5^ within the pharmaceutical industry. However, their efficacy would be compromised in this context. This traditional strategy prioritises safety at the expense of effectiveness, as the additional metabolism required to convert prodrugs into their active forms inevitably reduces the effective concentration. This issue has troubled scientists for many years. Compared to other adjuvants, ubiquitous lysozyme exhibits the broad-spectrum applicability across diverse patient populations, ensuring the safety and biocompatibility of our strategy in different clinical practices. More importantly, it has specific bacterial targeting, concentrating around bacteria while showing less enrichment around host cells. Such a gradient enhances the selective amplification of synergistic effects on pathogens. Therefore, our strategy empowers scientists to simultaneously achieve both enhanced safety (being microbiome-friendly in addition to being a prodrug) and improved efficacy (synergistic effect) in the pharmaceutical industry.

Overall, these findings provide many combination therapies for future clinical applications, along with an in-depth investigation of the mechanisms behind these combinations and novel functional insights into various oxazolidinone-class antibiotics against multi-drug resistant bacteria, such as *MRSA*, although larger cohorts in relevant studies would be more conclusive. Moreover, this study unveils a novel class of drug re-sensitisation approaches that position lysozyme as the representative adjuvant (independent of its well-known role as antimicrobial agent) for various oxazolidinone-class antibiotics with phosphorylation modifications, particularly TP, in the fight against broad-spectrum drug-resistant Gram-positive bacteria.

## Contributors

Qi Zhang, Yang Yang, Ying Yang, Jin Shang, Shan Su, and Xiao-Xiao Li carried out experiments. Qian Zhao and Qi Zhang designed the project and wrote the manuscript with the advice from Zhao Liu, Peng Gao, Richard Yi-Tsun Kao, Ben Chi-Bun Ko, and Benjamin Thompson.

## Data sharing statement

All data generated or analysed during this study are included in this published article and its supplementary information files.

## Declaration of interests

Qian Zhao and Qi Zhang have filed a patent application (US application No. 18/888,874) related to the work presented in this manuscript. All other authors declare no conflict of interest.
