# Supplementary material for: A strategy to re-sensitise drug-resistant Gram-positive bacteria to oxazolidinone-class antibiotics
Source: eBioMedicine. 2025 Sep 4;119:105914. doi: 10.1016/j.ebiom.2025.105914 (PMC12446384; doi:10.1016/j.ebiom.2025.105914)
Supplement: Figures S1–S8 [file mmc3.docx]

Supplementary Materials for

**A strategy to re-sensitize drug-resistant Gram-positive bacteria to oxazolidinone-class antibiotics**

Qi Zhang et al.

Corresponding author: Prof. Qian Zhao (email: q.zhao@polyu.edu.hk; telephone number: +852 34008711)

The file includes:

Title page 1

Supplementary Figures 1 to 82-9

Other material for this manuscript includes:

Supplementary Tables 1-3

**
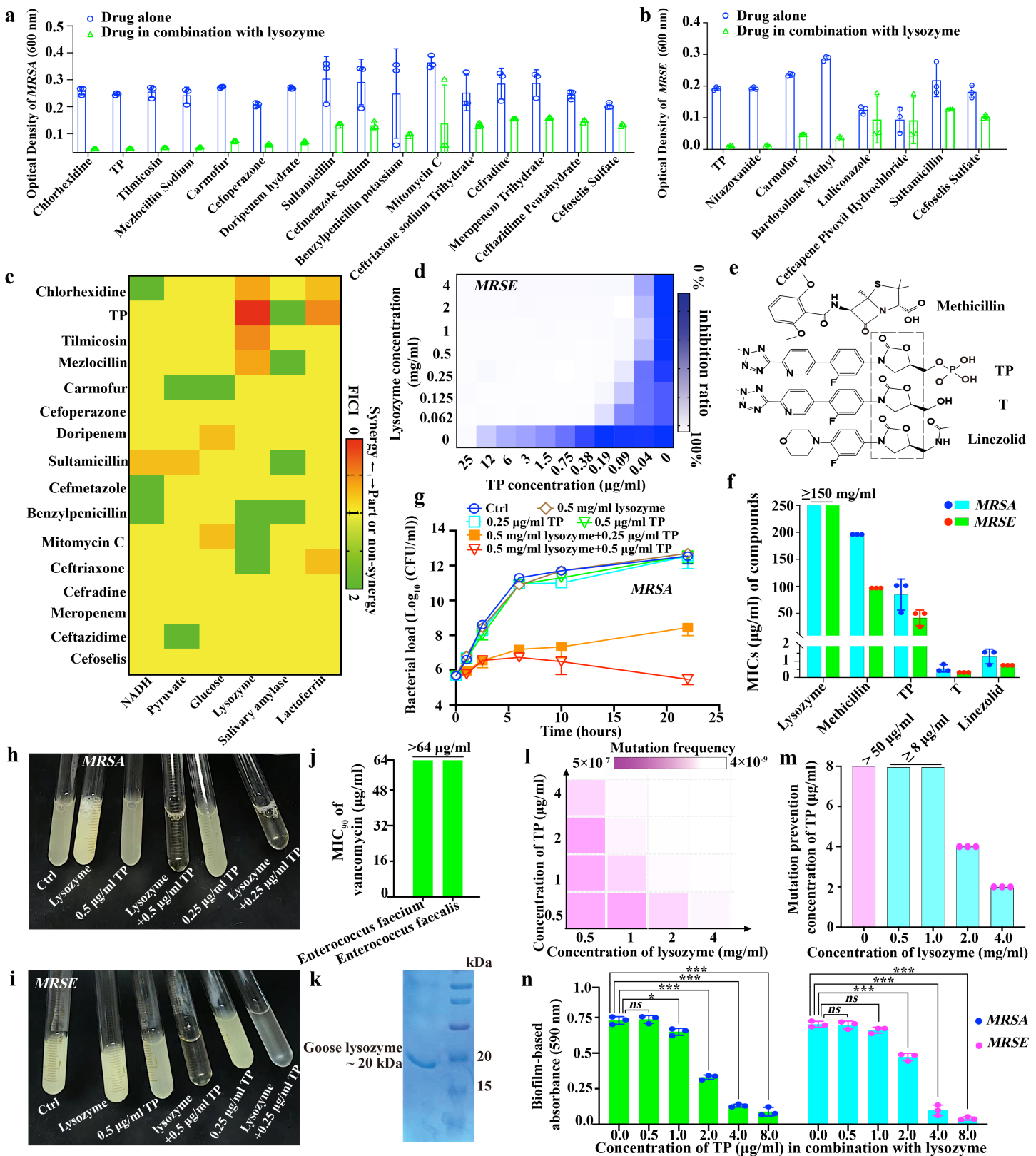
**

**Figure S1: Synergistic effect between TP and lysozyme *in vitro*. (a-b)** Optical densities at 600 nm of **(a)** *MRSA* or **(b)** *MRSE* treated with selected drugs in the absence and presence of 1·0 mg/ml lysozyme. Synergistic effect was defined as ≥90% inhibition ratio. **(c)** Representative heat map showing the cross-evaluation of the combined effects of natural metabolites and selected representative antibiotics, against *MRSA*. **(d)** A representative heat map showing the inhibited growth of *MRSE* under combination therapy. **(e)** Chemical structures of methicillin, TP, T and linezolid, and **(f)** their minimum inhibitory concentrations (MIC_90_) against *MRSA* or *MRSE*. **(g)** Representative time-kill curves for *MRSA* under different treatments over 22 hours and bacterial cultures of **(h)** *MRSA* or **(i)** *MRSE* at the end of experiments. **(j)** The MIC_90_ of vancomycin against *Enterococcus faecium* and *Enterococcus faecalis*. **(k)** Representative gel image showing the purity of G-type lysozyme extracted from Chinese geese (*Anser cygnoides*) eggs. Specifically, egg white was diluted in 50 mM PBS (pH 7·0), centrifuged (12,000 *g*, 15 minutes, 4°C), and subjected to isoelectric precipitation (pH 4·0, 6·0 and 7·0). After filtration (0·45 μm), the supernatant was purified by cation-exchange chromatography and size-exclusion chromatography. **(l)** Heat map showing mutation frequencies in *MRSE* and **(m)** bar plot showing mutation prevention concentrations of TP in the absence and presence of lysozyme. **(n)** Biofilm-based absorbance analyses in *MRSA* and *MRSE* under different treatments, in which bacterial growth was inhibited even after biofilm formation. Two-tailed Student's t-tests were applied with a significance threshold: ^*^*p*<0·05, ^**^*p*<0·01 and ^***^*p*<0·001. All assays were performed in triplicate (three independent replicates per group).

**
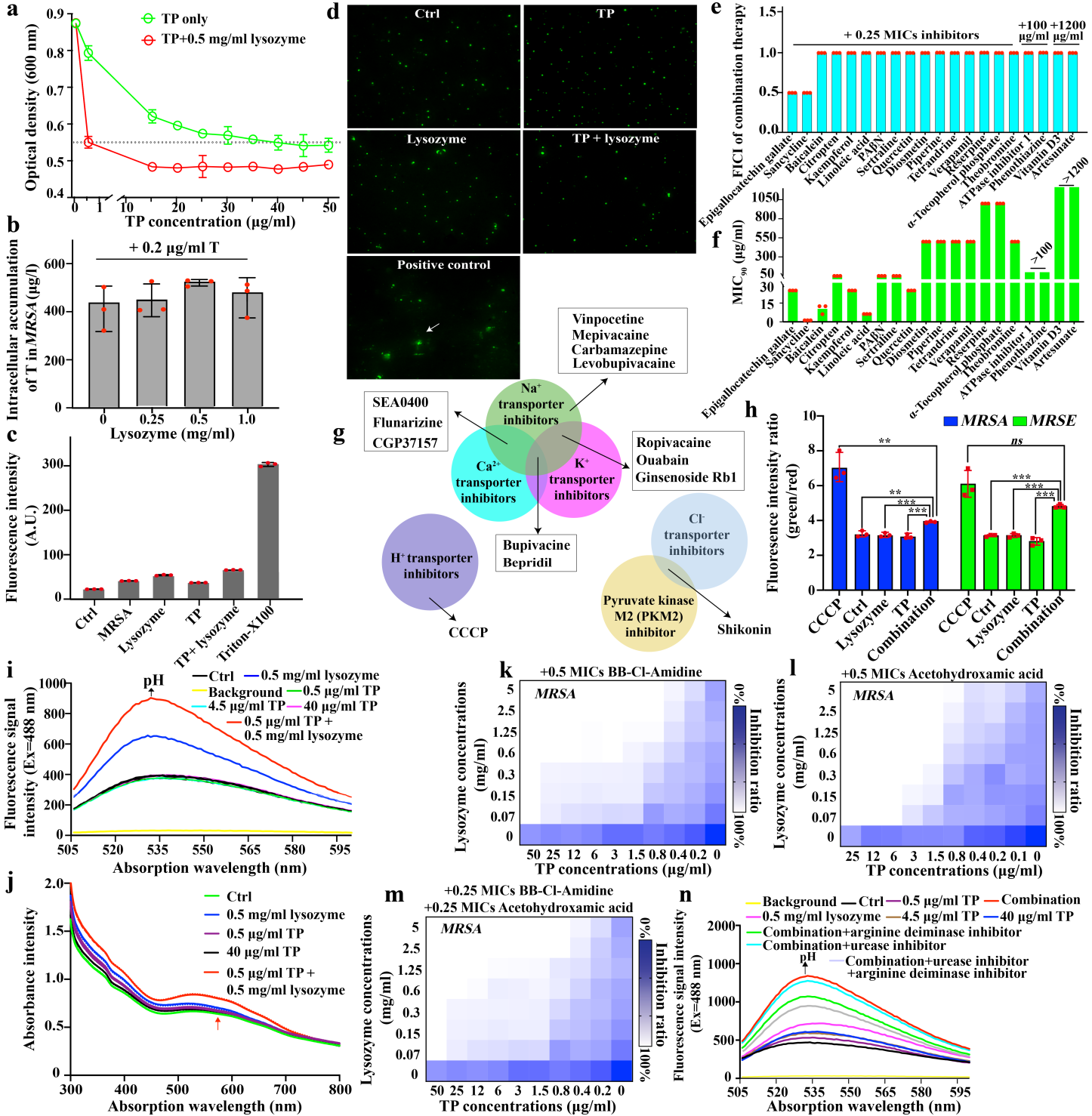
**

**Figure S2: Combination therapy alkalized cytoplasm.** **(a)** Optical densities of *MRSA* treated with T or TP in the absence or presence of 0·5 mg/ml lysozyme for one hour. **(b)** Intracellular accumulation of T in *MRSA* exposed to 0·2 μg/ml T in the absence or presence of lysozyme for 0·5 hour. **(c)** Permeability analysis of cell membrane of *MRSA* under different treatments. Here, bacterial membrane integrity was assessed via propidium iodide (PI) uptake (0·5 μM; λ_ex_=535 nm, λ_em_=615 nm) since PI penetrates only nonviable cell membranes. Log-phase *MRSA* was treated with TP (0·5 μg/ml) in the absence or presence of 0·5 mg/ml lysozyme for one hour. Those treated with 0·5% Triton X-100-treated and 1×PBS served as control groups. **(d)** Assessment of cell wall integrity in *MRSA* under different treatments. Bacteria similar to those used in permeability assay were embedded in agarose microgels, lysed (0·5% NP40 and 0·5% Triton-X100) and stained with 1×SYBR-Gold for fluorescence microscopy (representative images are shown). **(e)** FICIs of combination therapies involving 21 representative pumps inhibitors in conjunction with TP and **(f)** their minimum inhibitory concentrations (MIC_90_) against *MRSA*. **(g)** Classification diagrams of 14 ions-targeting transporter inhibitors. **(h)** Analyses of membrane potentials in *MRSA* and *MRSE* after one hour treatments. Mid-log phase bacteria were treated with monotherpy or combination therapy of lysozyme (0·5 mg/ml), TP (0·5 µg/ml), CCCP (5·0 μM) or 1×PBS, then stained with 30 µM DiOC_2_(3). Flow cytometry measured fluorescence ratios (green: 488 nm; red: 633 nm), with higher ratios (green/red) indicating weaker membrane potentials. **(i)** Alkalized cytoplasmic pH level in *MRSA* under different treatments. **(j)** Activation of alkaline phosphatase from *MRSA* in combination group resulted in more catalytic products (NBT-formazan). **(k-m)** Representative heat maps of *MRSA* under combination therapies with inhibitors against **(k)** arginine deiminase, **(l)** urease or **(m)** both enzymes. **(n)** The pH-dependent fluorescence signal intensities at 520 nm under different treatments. Two-tailed Student's t-tests were applied with a significance threshold: ^*^*p*<0·05, ^**^*p*<0·01 and ^***^*p*<0·001. All assays were performed in triplicate (three independent replicates per group).

**
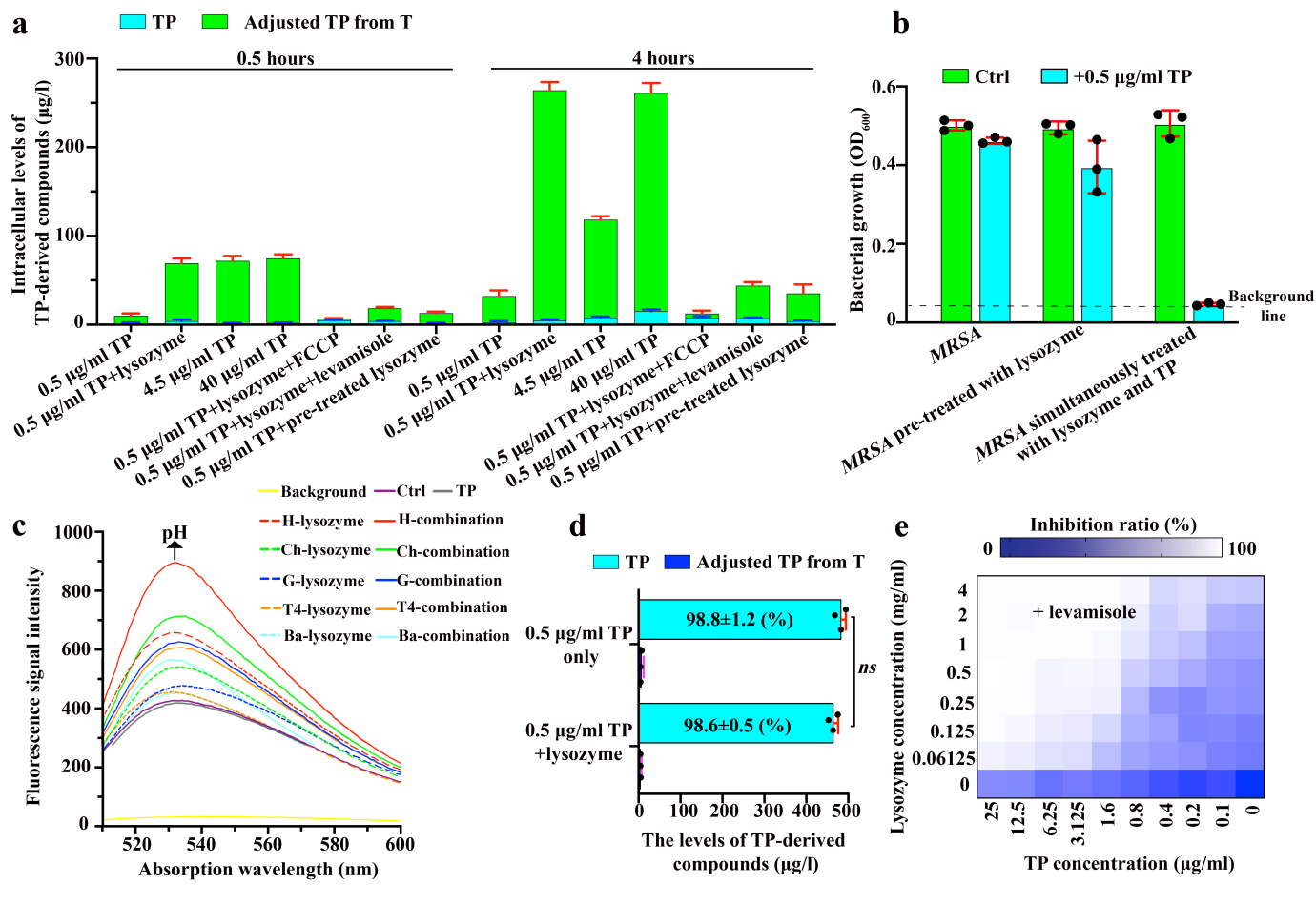
**

**Figure S3: By inducing cytoplasmic alkalization, the combination treatment promoted the conversion of TP to T.** **(a)** Stacked bar charts showing the intracellular levels of TP-derived compounds (TP and its active form T. Notably, the concentration of adjusted TP was calculated by multiplying the measured concentration of T resulting from TP-to-T conversion by the TP-to-T molecular weight ratio) in log-phase *MRSA* under different treatments at 37 °C. As the processing time increased (from half to four hours), the intracellular levels of TP-derived compounds rose to varying degrees across all groups, with the combination group exceeding the saturation threshold of 4·5 μg/ml, reaching concentrations comparable to those in the 40 μg/ml group. However, this increase could be suppressed by FCCP (a cytoplasmic acidifier) and levamisole (an ALP inhibitor), although the inhibitory effect of the latter was weaker than that of FCCP. Bacteria in lysozyme pre-treatment group were pretreated with 0·5 mg/ml lysozyme to remove cell walls (if applicable), washing away the excess lysozyme followed by TP treatment. Notably, most of the TP-derived compounds were T converted from TP. **(b)** The OD_600_ of bacterial overnight culture showed that *MRSA* remained viable unless lysozyme and TP were applied simultaneously. **(c)** Cytoplasmic pH levels in *MRSA* treated with 0·5 μg/ml TP in the presence or absence of a specific lysozyme (0·5 mg/ml) for 0·5 hours at 37 °C. Notably, human lysozyme (H-lysozyme) showed the strongest cytoplasmic alkalinization ability. Lysozymes from chicken, goose, T4 bacteriophage, and *Bacillus subtilis* were labeled as Ch-lysozyme, G-lysozyme, T4-lysozyme, and Ba-lysozyme (combinations named analogously), respectively. **(d)** Lysozyme did not demonstrate the ability to hydrolyze TP *in vitro*. Specifically, 0·5 μg/ml TP was incubated with or without 0·5 mg/ml lysozyme for 0·5 hours at 37 °C, followed by LC-MS/MS analysis. Regardless of treatment conditions, the *in vitro* data consistently showed that TP constituted the predominant form of TP-derived compounds, with no significant difference. Two-tailed Student's t-tests were applied with a significance threshold: ^*^*p*<0·05, ^**^*p*<0·01 and ^***^*p*<0·001. **(e)** A representative heatmap displaying the growth of *MRSA* under different treatments. Synergistic effect between TP and lysozyme was significantly attenuated when an ALP inhibitor levamisole was added. All assays were performed in triplicate (three independent replicates per group).

**
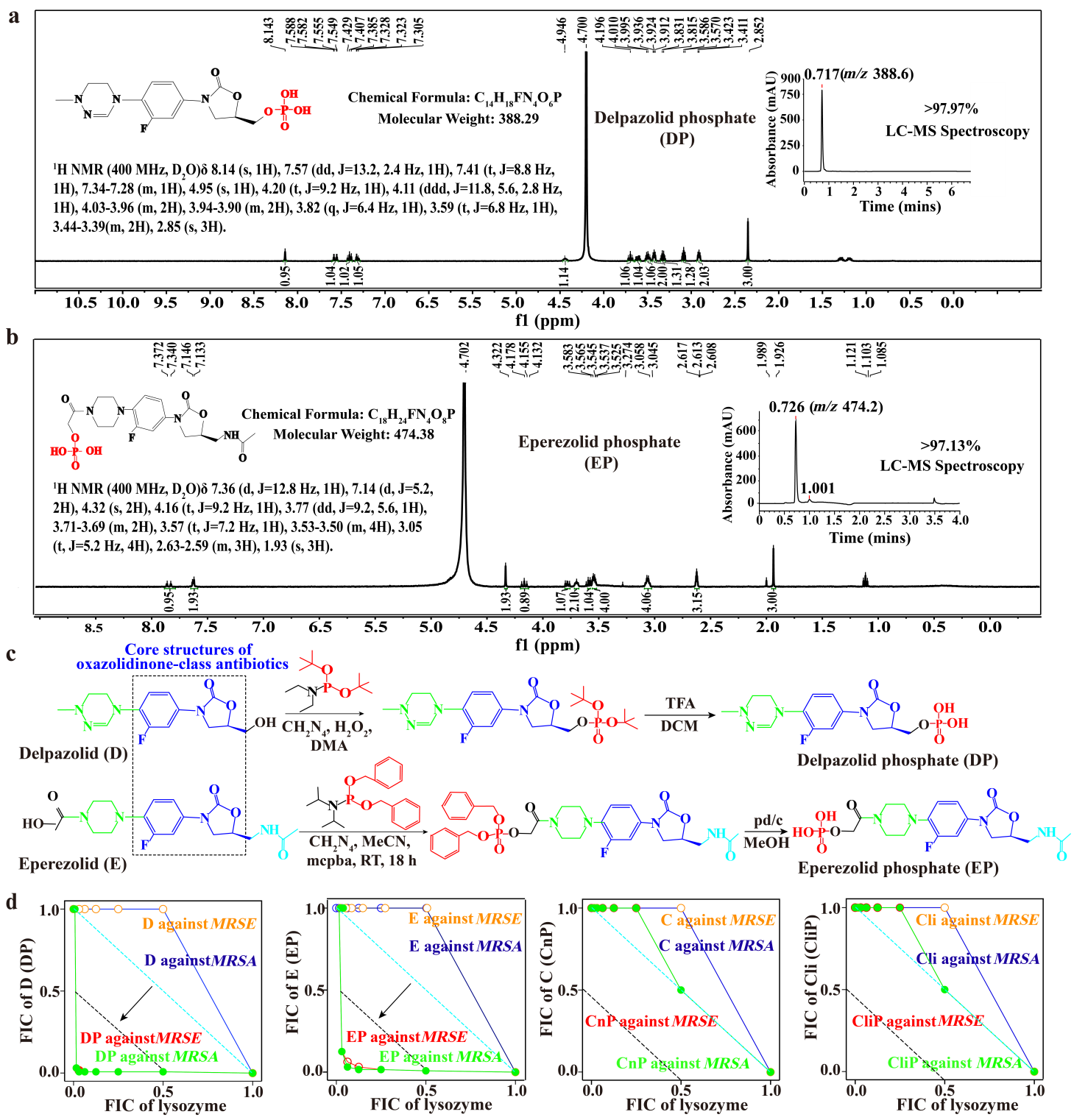
**

**Figure S4: Chemical syntheses of several oxazolidinone-class antibiotics and their bio-activities.** The spectra of synthesized **(a)** delpazolid phosphate and **(b)** eperezolid phosphate using NMR and LC-MS (insets), showing their validities and purities. **(c)** Synthesis routes of DP and EP, and their structural comparison. **(d)** Isobolograms of combination therapies consisting of lysozyme and several antibiotics against *MRSA* or *MRSE*. The dash lines indicated ideal isobole, where drugs acted additively and independently. Data points below this black dash line indicated synergism. D: delpazolid; DP: delpazolid phosphate; E: eperezolid; EP: eperezolid phosphate; C: contezolid; CnP: contezolid phosphoramidic acid; Cli: clindamycin; CliP: clindamycin phosphate.

**
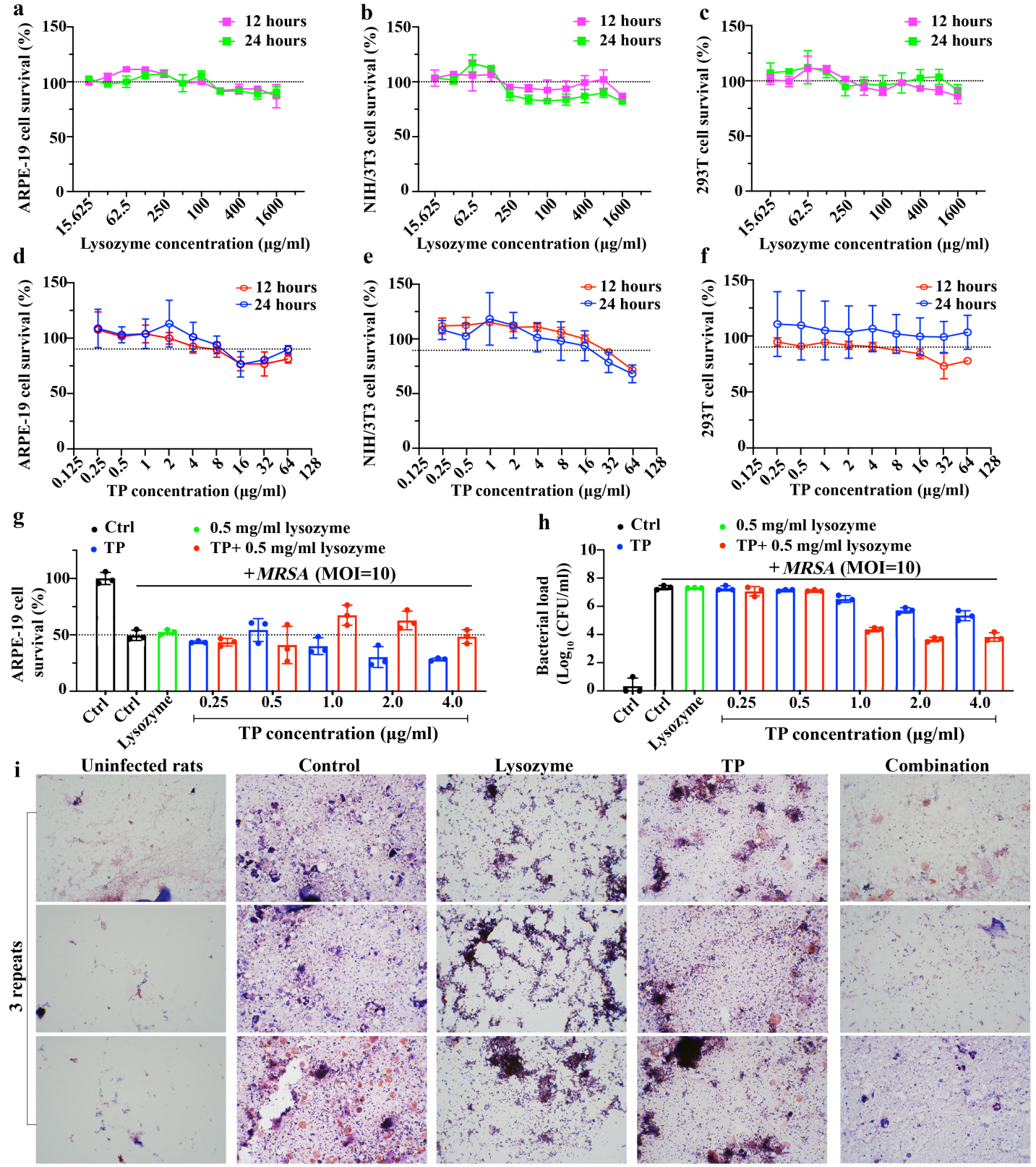
**

**Figure S5: TP demonstrated the strong synergistic effect with lysozyme in cell-based infection model and a rat skin infection model.** Survival ratios of **(a, d)** ARPE-19 cells, **(b, e)** NIH/3T3 cells and **(c, f)** 293T cells exposed to lysozyme or TP for 12 and 24 hours. These cells (50% confluency) were treated with lysozyme, TP or some oxazolidinone analogs (delpazolid/eperezolid and their phosphorylated forms) at varying concentrations at 37 °C in 5% CO_2_-humidified atmosphere. Cell count without drug treatment was normalized for 100% survival ratio. All cells were from our laboratory stock and and authenticated by PCR. **(g)** Survival ratios of ARPE-19 cells and **(h)** cell-associated bacterial loads after infection with mid-log-phase *MRSA* at a multiplicity of infection (MOI) of 10·0 for 20 hours. Here, ARPE-19 cells (as host cells, 1·0×10^4^/well) were infected with *MRSA* at 37 °C for three hours. After PBS washing, infected cells were treated with TP, lysozyme, or their combination for 24 hours. Cell survival was assessed by CCK-8 assay (absorbance was measured at 450 nm, with 630 nm as a reference), calculated as: (Abs_sample_-Abs_negative control_)/(Abs_positive control_-Abs_negative control_)×100%, where medium in the absence of cells served as the negative control, while medium in the presence of cells served as the positive control. Bacterial loads were quantified by lysing the ARPE-19 cells with PBS containing 1% Triton X-100 and serially diluting the resulting lysates onto LB-agar plates. **(i)** Representative Gram-staining images of *MRSA* in rat wounds under different treatments, indicating that combination therapy significantly reduced bacterial loads in infected wounds. Each blue point represented a bacterium. Randomly selected images per group were shown. All assays were performed in triplicate (three independent replicates per group).

**
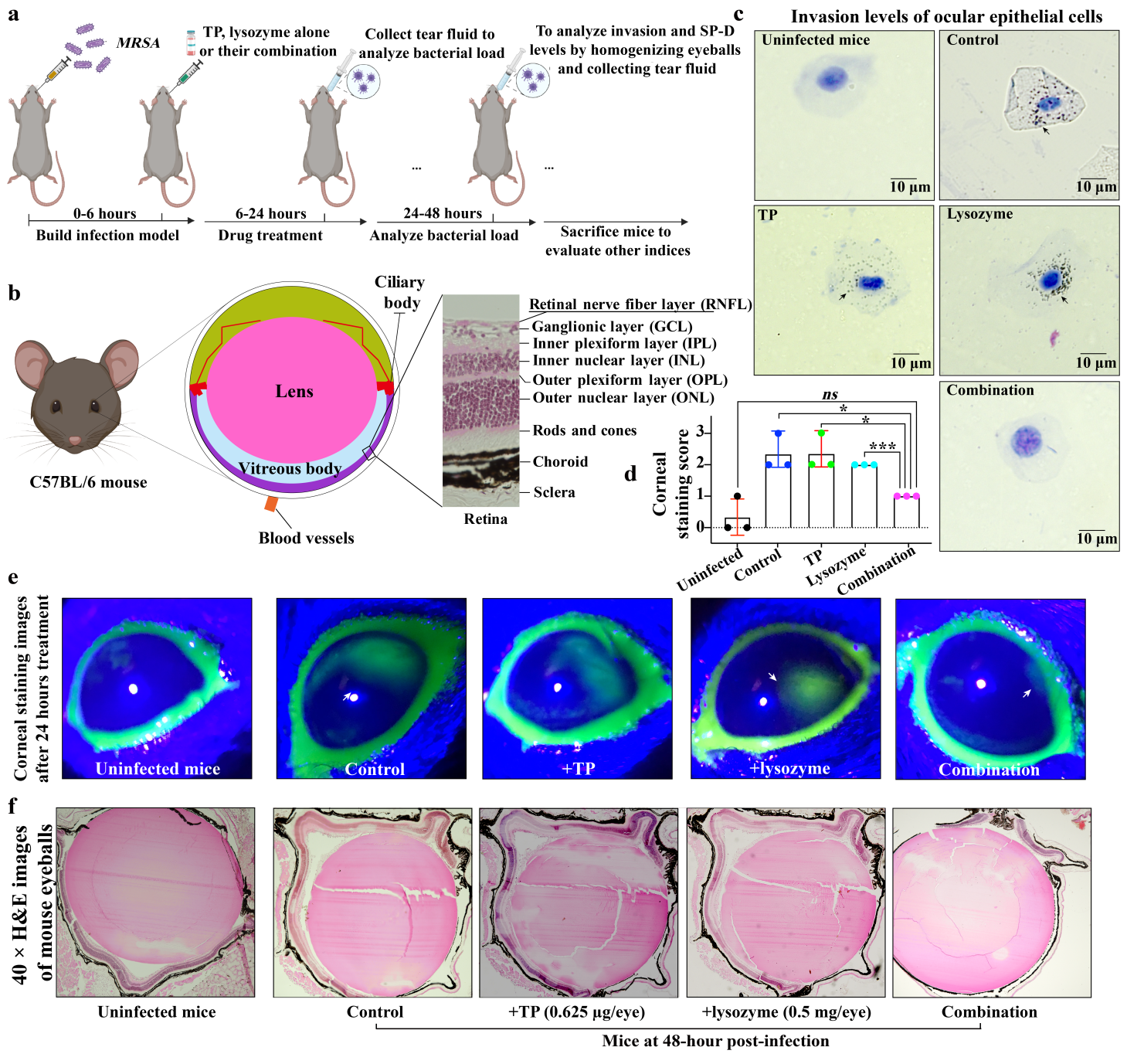
**

**Figure S6: Successful observation of combination effect in ocular bacterial infection model. (a)** Flow chart detailing the infected mice in the ocular infection model. **(b)** Mouse ocular structure, in which some substructures including the retina were labeled. **(c)** Representative images of epithelial cells in tears of mice under different treatments. Black arrows indicated invading bacteria. **(d)** Normalized injury scores of eyes of mice under different treatments for 24 hours and **(e)** their representative fluorescein images. White arrows indicated damaged corneal integrity. (**f**) Representative H&E staining images (40×) of eyeballs from C57BL/6 mice under different treatments. Two-tailed Student's t-tests were applied with a significance threshold: ^*^*p*<0·05, ^**^*p*<0·01 and ^***^*p*<0·001. All assays were performed in triplicate (three independent replicates per group).

**
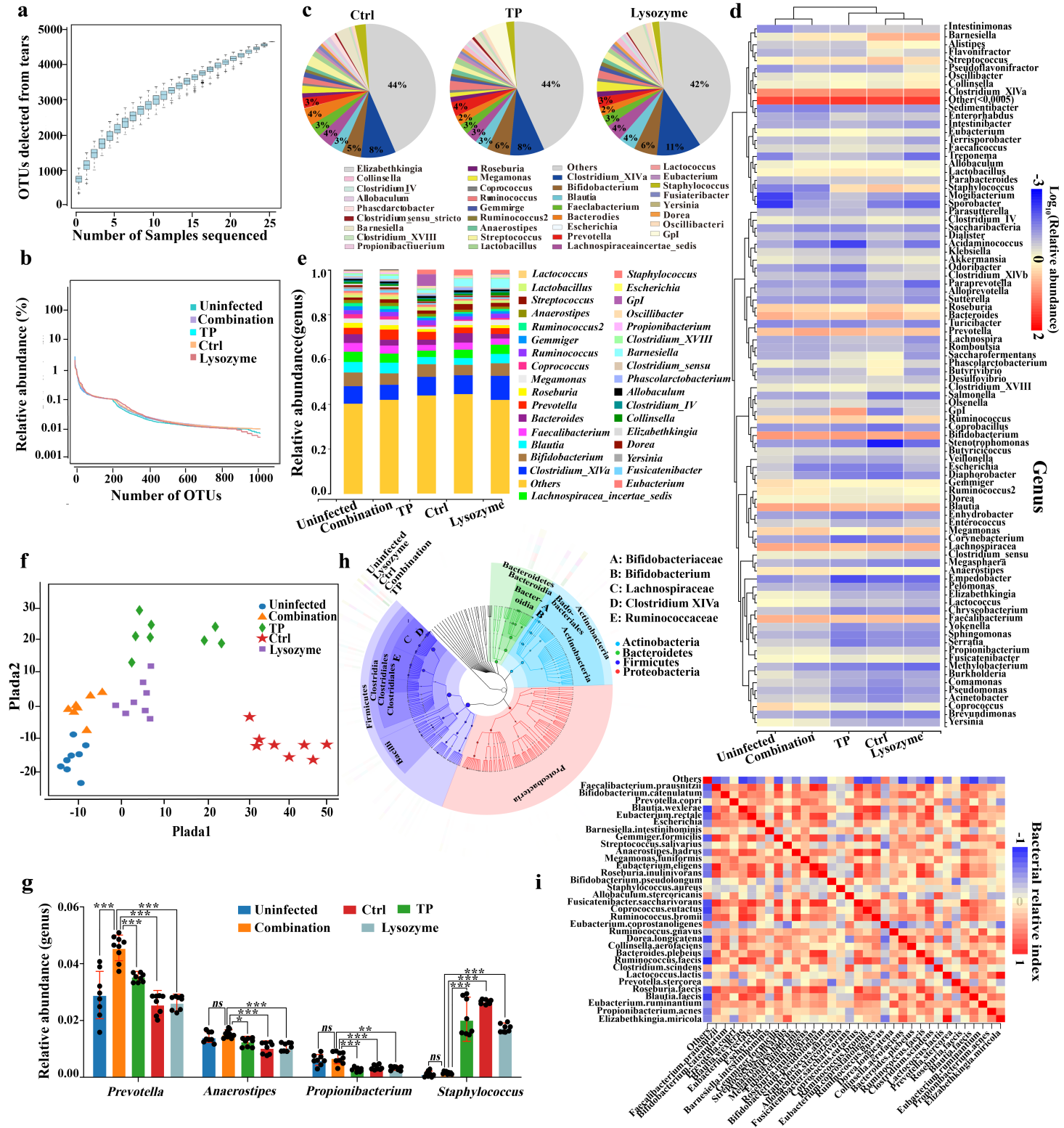
**

**Figure S7: Bacterial microbiome in tears of C57BL/6 mice under different treatments. (a)** Species accumulation curves and **(b)** OTU rank curves for bacterial communities in tears by using 16S rRNA analysis. **(c)** Composition analyses of bacterial communities in tears from infected mice without treatment (*left*), treated with TP (*middle*) or lysozyme (*right*). Inserted values indicated corresponding composition percentages. Herein, *Staphylococcus* (*MRSA*) was highlighted in orange yellow. **(d)** Heat-maps representing the relative abundance of detected bacterial genera, demonstrating that combination therapy effectively restored bacterial communities in tears. **(e)** Columns to compare bacterial compositions in tears of mice under different treatments. Bacteria were also described at the genus level. **(f)** Partial least squares-discriminant analysis of bacterial communities. **(g)** Changes in proportions of several bacteria in tear communities from mice under different treatments, revealing that combination therapy not only decreased pathogens including *Staphylococcus*, but also increased some beneficial commensal probiotics including *Prevotella*, *Propionibacterium* and *Anaerostipes*. **(h)** Graphlan-map displaying evolutionary branching trees of detected genera. The heat-cycles in outer layers showed relative genus-associated abundances in different groups highlighted by different colors. **(i)** Heat map illustrating correlation coefficient among different genera. Herein, red and blue indicated co-existence and inter-exclusion, respectively. Two-tailed Student's t-tests were applied with a significance threshold: ^*^*p*<0·05, ^**^*p*<0·01 and ^***^*p*<0·001. Assays were conducted with either three, five, or eight repetitions (three, five, or eight independent replicates per group).

**
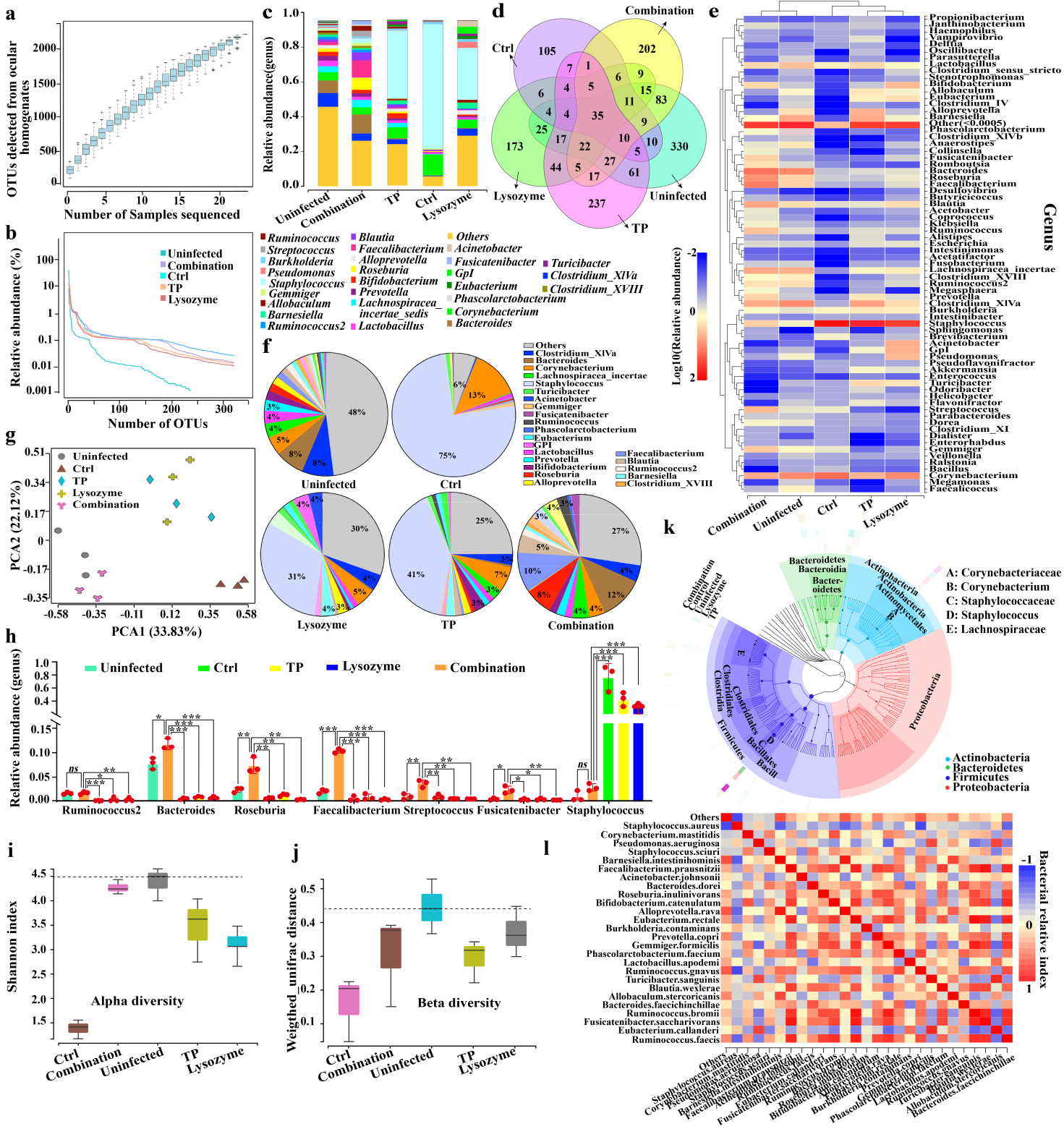
**

**Figure S8: Microbiome of mice ocular homogenates. (a)** Species accumulation, **(b)** OTU rank curves for bacterial communities and **(c)** bacterial composition analysis in ocular homogenates. **(d)** Venn diagram showing detected operational taxonomic units (OTUs) in ocular homogenates of mice under different treatments. 35 core OTUs were shared among different groups. **(e)** Heat map showing the relative abundance of detected bacterial genera in ocular homogenates of mice under different treatments. **(f)** Composition analysis of bacterial communities in ocular homogenates from healthy mice, infected mice without treatment, and infected mice treated with TP, lysozyme, or combination therapy. **(g)** Principal component analysis and **(h)** representative bacteria with changing proportions (genus level) of bacterial communities in ocular homogenates. **(i)** Alpha and **(j)** beta diversity analyses of bacterial communities in ocular homogenates, with bottom-top lines of diversity boxplot indicating minimum, median and maximum values, respectively. **(k)** Graphlan map displaying evolutionary trees of detected genera. **(l)** Heat map illustrating co-existence (red) and inter-exclusion (blue) among different genera. Two-tailed Student's t-tests were applied with a significance threshold: ^*^*p*<0·05, ^**^*p*<0·01 and ^***^*p*<0·001. Assays were conducted with either three, five, or eight repetitions (three, five, or eight independent replicates per group).
